# INO80/SWR remodelers regulate Pol II transcription through BRD2 and chromatin landscape

**DOI:** 10.1093/nar/gkaf892

**Published:** 2025-09-17

**Authors:** Meitong Liu, Qiqin Xu, Hui Wang, Xiong Ji

**Affiliations:** State Key Laboratory of Gene Function and Modulation Research, Key Laboratory of Cell Proliferation and Differentiation of the Ministry of Education, Beijing Advanced Center of RNA Biology (BEACON), School of Life Sciences, Peking-Tsinghua Center for Life Sciences, Peking University, Beijing 100871, China; State Key Laboratory of Gene Function and Modulation Research, Key Laboratory of Cell Proliferation and Differentiation of the Ministry of Education, Beijing Advanced Center of RNA Biology (BEACON), School of Life Sciences, Peking-Tsinghua Center for Life Sciences, Peking University, Beijing 100871, China; State Key Laboratory of Gene Function and Modulation Research, Key Laboratory of Cell Proliferation and Differentiation of the Ministry of Education, Beijing Advanced Center of RNA Biology (BEACON), School of Life Sciences, Peking-Tsinghua Center for Life Sciences, Peking University, Beijing 100871, China; Agricultural Animal Diseases and Veterinary Public Health Key Laboratory of Sichuan Province, Key Laboratory of Agricultural Bioinformatics, Ministry of Education, Sichuan Agricultural University, Chengdu 611130, China; State Key Laboratory of Gene Function and Modulation Research, Key Laboratory of Cell Proliferation and Differentiation of the Ministry of Education, Beijing Advanced Center of RNA Biology (BEACON), School of Life Sciences, Peking-Tsinghua Center for Life Sciences, Peking University, Beijing 100871, China

## Abstract

INO80/SWR remodelers, including INO80, P400/TIP60, and SRCAP, have been viewed to regulate H2A.Z dynamics, but how they control specific gene expression remains unclear. Here, we reveal distinct phenotypes arising from short- and long-term perturbations following the depletion of INO80/SWR remodelers. We identified the direct target genes of INO80, P400, and SRCAP remodelers. Mechanistically, we found that INO80/SWR remodelers interact and facilitate the chromatin occupancy of the H2A.Zac reader protein BRD2, thereby elucidating the regulation of Pol II transcription by INO80/SWR remodelers. Additionally, while the degradation of P400 or SRCAP led to a reduction in H2A.Zac, INO80 depletion did not affect H2A.Zac occupancy but decreased the occupancy of P400 and SRCAP. Collectively, our findings suggest that INO80/SWR remodelers primarily regulate the chromatin landscape and influences BRD2 chromatin binding, thereby shedding light on their mechanisms in Pol II transcription regulation during development and disease.

## Graphical Abstract

**Graphical Abstract:**
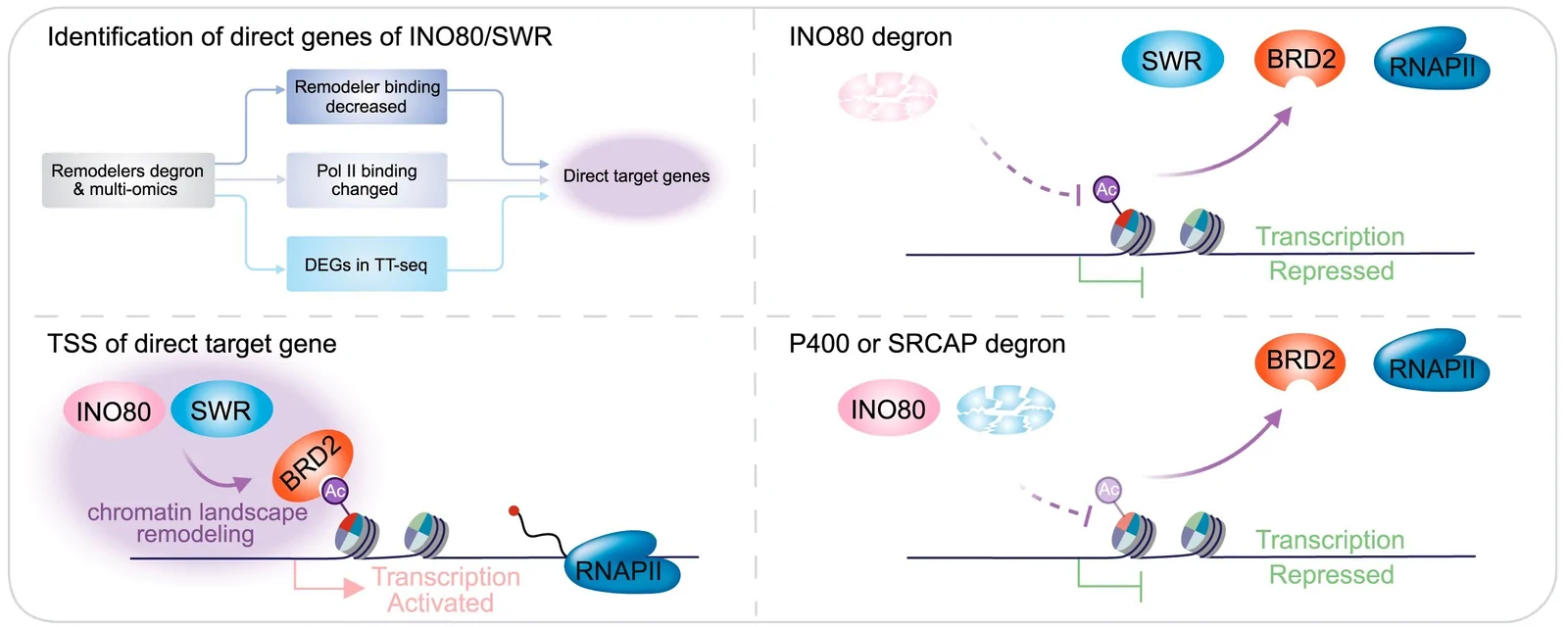

## Introduction

Pol II occupies gene promoters, overcomes chromatin barriers like the +1 nucleosome, initiates, elongates, and terminates transcription in mammalian cells [1–13]. The incorporation of H2A.Z into the +1 nucleosome can contextually modulate Pol II transcription [14–22]. Some transcription factors recruit chromatin remodelers to sequence-specific elements [23, 24]. ATP-dependent chromatin–remodeling complex regulates chromatin dynamics to enable specific gene expression programs during development and disease [11, 25–27]. Understanding the molecular mechanisms that govern the regulation of Pol II transcription by H2A.Z-related chromatin remodelers in mammalian cells is of utmost importance. These insights are particularly significant as they can be broadly applied to connect the chromatin landscape with specific gene expression programs during both development and diseases.

RUVBL1/2 are shared subunits of INO80/SWR remodelers (INO80, P400, and SRCAP). Our previous work demonstrated that RUVBL2 directly regulates Pol II condensates at promoter-proximal regions [28], independent of the INO80/SWR remodelers. In this work, we aimed to investigate the transcriptional regulation by INO80/SWR remodelers using acute protein degradation followed by multi-omics analyses. The INO80 and SWR1 remodelers (INO80/SWR) consist of the INO80 and SWR1 subfamilies, comprising the SRCAP and P400/TIP60 complexes [29]. INO80 remodelers seem to play a role in evicting and mobilizing H2A.Z; however, this remains a controversial topic [30–32]. SWR1 remodelers are associated with the incorporation and acetylation of H2A.Z [31, 33–40]. Previous investigations of INO80/SWR remodelers have predominantly relied on long-term RNAi or mutagenesis approaches, as well as *in vitro* assays, and showed that the involvement of these remodelers in cell-type-specific gene expression regulation [41–48]. While acute protein degradation techniques have been widely utilized [49, 50], the specificity and potential off-target effects of these methods have not yet undergone comprehensive examination. As a result, the immediate roles of INO80, P400, and especially SRCAP in endogenous chromatin structures, Pol II transcription, and their functional relationships have not been thoroughly elucidated.

There is growing interest in the functions of multi-subunit enzymatic complexes [51–53]. For example, Pol II, which consists of 12 subunits [54], gains diverse functions through specialized subunits in addition to its canonical role in RNA synthesis [55, 56]. The INO80/SWR remodelers are also multi-molecular complexes [57–59]. Previous studies have mostly investigated their roles in chromatin structures [34, 37, 60], and only a portion of the complex is directly involved in ATP-dependent chromatin remodeling activity. Little is known about the transcriptional functions of the complex.

In this study, we investigated the immediate effects of depleting INO80, P400, and SRCAP chromatin remodelers on Pol II transcription in mESCs. We identified the direct target genes of INO80, P400, and SRCAP by integrating TT-seq experiments with analyses of remodeler binding and Pol II occupancy. Our findings indicate that the loss of INO80/SWR remodelers results in distinct changes to the chromatin landscape, including alterations in H2A.Z acetylation, H3.3 incorporation, and the binding of other INO80/SWR remodelers. Furthermore, our results suggest that INO80/SWR remodelers modulate the chromatin landscape, influencing BRD2 chromatin binding and thereby underlying their mechanisms of Pol II transcription regulation. TT-seq after BRD2 degradation demonstrated substantial overlap between BRD2-dependent and remodeler-dependent genes. H2A.Z/H2A.Zac CUT&Tag showed increased chromatin occupancy upon BRD2 depletion, while INO80/SWR CUT&Tag after BRD2 degradation indicated decreased INO80 occupancy, increased P400 occupancy, and stable SRCAP occupancy. These findings suggest a potential regulatory circuit coordinating BRD2 chromatin occupancy, INO80/SWR remodeler function, and H2A.Z modification states.

## Materials and methods

### Cell culture

Mouse embryonic stem cells (mESCs) were cultured in 2i/mLIF medium as depicted before [28], which composed of DMEM containing 1 mM L-glutamine, 1 × nucleosides (Sigma, ES-008), 1 × nonessential amino acids (Milipore, TMS-001-C), 1 × sodium pyruvate (Gibco, 11 360 070), 1 × penicillin/ streptomycin (Gibco, 15140–122), 0.1 mM β-mercaptoethanol (Sigma, M3148), 3 μM CHIR99021 (Selleck, S1036), and 1 μM PD0325901 (Selleck, S1263), 1000 U/ml mouse leukemia inhibiting factor (mLIF) (Milipore, ESG1107), and also supplemented with 15% fetal bovine serum (FBS) (VivaCell, 04–001–1A), incubated at 37°C with 5% CO_2_ in a humidified incubator. HEK293T cells were cultured in DMEM (Gibco, C11995500BT) supplemented 8% FBS and 1 × penicillin/ streptomycin. Drosophila S2 cells were maintained in insect culture medium R69007 addition with 10% FBS and 1 × penicillin/streptomycin. For target protein degradation, the degron cells were firstly induced with 1 μg/ml Dox in the 2i medium to express the OsTir1 for 12 h, then 500 μM auxin were added to induced the degradation for 1–24 h, then cells were treated or harvested for next assays.

### Cell lines generation

For INO80, P400 and SRCAP degron, Tet-ON-hPGK-OsTIR1 (V6.5) mESC cells were transfected with the equal amount of mAID-GFP-NeoR donor fragment and *C*-terminal region gRNA, respectively, using FuGENE HD transfection reagent in 6 wells plates. The sequences of gRNAs see Supplementary Table S7. Two day later, cells were expanded to 10 cm dish and added 500 μg/ml Geneticin to the medium, then changed medium freshly every day to remove the dead cells, picked up the clones after 7–10 days later and carried out the genotyping. The homologous clones were further sanger sequenced and immunoblot validated. To get the Pol II-TurboID cell lines, the RPB1 TurboID-HygroR donor fragments and the *C*-terminal gRNA were co-transfected into the INO80, P400, and SRCAP degrons, respectively, and screened using 200 μg/ml Hygromycin B as same as described above. The confirmed cell lines were stored in to liquid nitrogen.

### Western blot

Samples were harvested in 1.5 × loading buffer and boiled for 10 min, then loaded on 8 or 12% (w/v) sodium dodecyl sulfate-polyacrylamide gel electrophoresis (SDS-PAGE). The fractionated proteins were transferred onto methanol pre-actived PVDF membranes together with a visible protein marker through 300 mA for 2 h in ice bath, for high molecular weight molecules, 0.1% SDS were extra supplemented into the Tris-Gylcine transfer buffer. Membranes were blocked with 5% (w/v) skim milk/ TBST (1‰ Tween-20) for 1 h at room temperature (RT) and then incubated with indicated the primary antibodies (diluted according to manufacturer's instruction) overnight at 4°C. After three times washing with TBST, membranes were incubated in corresponding horseradish peroxidase-labeled secondary antibody (diluted in TBST with 1% BSA following the manufacturer’s instruction) for 2 h at RT. Washed 5 times in TBST, and finally the reactive bands were visualized using ECL kit (Co Win Biotech Co., Ltd., Beijing) and captured with G.E AI 600 RGB imaging system. Pictures were further analyzed using ImageJ.

### ChIP-seq and ChIP-MS

ChIP-seq and ChIP-MS were performed as described previously [61]. Briefly, the pretreated cells were harvested by trypsinization following 1% formaldehyde (final concentration, wt/vol) crosslinked and stored at -80°C for downstream experiments. For chromatin immunoprecipitation, 20 million crosslinked cells (5% HEK293T cell were added as spike-in) were lysed gently for chromatin isolation with ice-cold NP-40 lysis buffer and glycerol / urea buffer, finally centrifugated at 12 000 rpm for 1 min at 4°C, the pellet of chromatin were resuspended in 1 ml sonication buffer, 1000 U MNase (NEB, M0247) and 5 mM CaCl_2_ were added and incubated for 15 min at 37°C with 750 rpm shaking. After quenched by 20 μl 0.5 M EDTA and 40 μl 0.5 M EGTA in ice bath, the chromatin was sonicated using Qsonica Q800R3 DNA Shearing Sonicator for 15 min with 60% AMPL. Soluble chromatin was isolated by centrifugation at 12 000 rpm for 10 min at 4°C, 20 μl supernatant were sampled as input, the rest supernatant was transferred into 2 new DNase free tube for immunoprecipitation. 1 μl GFP abs (ABcam, ab290), 1 μg RPB1 NTD abs (CST, 14958S),1 μl BRD2 abs (Proteintech, 22236–1-AP), and equal isotype IgG control for each ChIP reaction, respectively. After incubation overnight at 4°C with low-speed rotation, 30 μl Protein G magnetic beads were added into the mixture to capture for 4 h, then washed beads once with sonication buffer, twice with high-salt wash buffer, twice with LiCl wash buffer and finally three times with TE buffer. For ChIP-seq, beads were eluted and then decrosslinked with 4 μl proteinase K (Sigma, 20 mg/ml) at 65°C overnight, DNA was purified through the Tris saturated equilibrium phenol (phenol: chloroform: isoamyl alcohol (25: 24: 1) (pH8.0). ChIP-qPCR was carried out to check the enrichment, and the ChIP library was prepared following the instruction of VAHTS Universal Pro DNA Library Prep Kit for Illumina (Vazyme, ND608-01), and finally subject to Nova PE150 sequencing (AnoRoad, Beijing). For ChIP-MS, the beads and input were directly boiled twice using the 2 × SDS-PAGE loading buffer for 12 min [61], then the samples were loaded to SDS-PAGE, Coomassie Brilliant Blue stained gel samples were subjected to Orbitrap Fusion Lumos Tribrid Mass Spectrometer analysis.

### CUT&Tag

The INO80, P400 and SRCAP binding and H2A.Z, H2A.Zac, H3.3, INTS5 changes were examined using CUT&Tag experiment. According to the manufacture of Hyperactive Universal CUT&Tag Assay Kit for Illumina (Vazyme, TD903-01), simply, cells were slightly crosslinked with 0.1% formaldehyde for 2 min, and then absorbed on the ConA beads, and then incubated for primary abs in the antibody buffer with digitonin overnight at 4°C, corresponding secondary abs were incubated in the Dig-wash Buffer for 1 h at RT. Then washed beads/cells three times with Dig-wash Buffer, and next incubated the pA/G-Tnp for 1h at RT with low-speed rotation. After three times wash, the fragmentation buffer was added into the beads/cells for incubation 1 h at 37°C, finally the fragmented DNA was extracted using the DNA extract beads, and the library were amplified using the Illumina primers. The 200–500 bp size DNA were recovered for Nova PE150 sequencing analysis.

### qPCR

Prepare the Mix by combining 5 μL of 2 × Taq Pro Universal SYBR qPCR Master Mix, 0.4 μL of mixed forward and reverse primers (concentration 10 μM), and 3.6 μL of water, then mix well and keep in the dark on ice. Add 9 μL of the above Mix to each well of a 96-well plate, and then add 1 μL of DNA sample. Cover the 96-well plate with a transparent film, centrifuge at 2000 rpm for 2 min, and then place it in the BIO-RAD CFX Connect Real-Time polymerase chain reaction (PCR) instrument for the reaction.

### Chromatin-associated RNA-seq (ChAR-seq) and RNA-seq

To examine nascent transcriptome changes, the chromatin RNAs were isolated and examined. Briefly, the pretreated cells without cross-link were used to chromatin isolation as described above, then the native chromatin were extracted twice using Trizol reagent to obtain the chromatin associated RNA. After checked the quantity and quality and poly(A) RNA depletion, the ChAR-seq RNA library were generated using the no coding RNA library method with random primers and sequencing analyzed using Nova PE150 platform. For RNA-seq, degron cells were induced using auxin for 24 h, then cells were harvested for total RNA extraction. Simply, cells were lysed directly in Trizol reagent and extracted RNA according to the manufacturer's instruction, after examination the concentration and integrity of the total RNA, samples were delivered for poly(A) RNA library generation by Novogene Com., Beijing, and HiSeq Xten PE150 sequencing (Novogene, Beijing), two replicates were performed.

### ATAC-seq

To examine the chromatin accessibility after the INO80, P400 and SRCAP degradation, ATAC-seq were performed as depicted before [62] and following the TruePrep DNA Library Prep Kit V2 for Illumina (Vazyme, TD501). Around 50 000 viable cells were collected for penetrated with 0.1% NP40, 0.1% Tween-20, and 0.01% Digitonin in 50 μl cold RSB buffer, then fragmentated and tagged using the Tn5 transposase according to the kit manufacture for 30 min at 37°C in a thermomixer with 1000 rpm mixing. Reaction was stopped by addition 250 μl (five volumes) of DNA binding buffer, and extracted DNA according to the DNA extraction kit's instruction. The eluted DNA were amplified eight cycles for library generation using the Illumina primers, and 200–500 bp fragments were selected for sequencing analysis.

### Transient transcriptome sequencing (TT-seq)

To determine the gene expression profiles at the nascent RNA level in INO80, p400 and SRCAP degron mESCs, we performed TT-seq as described with some modifications [63–66]. Briefly, two 15-cm-dish mESCs treated with or without 1 h IAA were metabolically labeled with 1 mM 4sU (Macklin #T820213) for 30 min, and 10% of Drosophila S2 cells treated with 100 μM 4sU for 12 h were mixed as spike-in. Total RNA was extracted using TRIzol reagent according to the manufacturer's instructions. For RNA fragmentation, 500 μg RNA was fragmented with sonication under the condition of 10 cycles with 30 s on/30 s off at high energy (Diagenode Bioruptor plus). Denature total fragmented RNA at 65°C for 10 min, then place on ice for 5 min.Add 6 μL of biotin buffer(833 mM Tris-HCl (pH = 7.4) , 83.3 mM EDTA) and 100 μL of 0.2 mg/ml MTSEA biotin-XX linker (5x MTSEA-Biotin into 1x with DMF) to the 400 μL of fragmented RNA and mix well.Incubate the biotinylation reaction at RT for 30 min in the dark. The biotinylated RNA was extracted with phenol/chloroform/isoamyl alcohol (25:24:1) and enriched with Dynabeads™ M-280 (Thermo Fisher Scientific #11205D) following the manufacturer’s instructions. After extraction with TRIzol reagent, the purified RNA was used for library preparation with a VAHTS Universal V8 RNA-seq Library Prep Kit (Vazyme #NR605) according to the manufacturer’s manual and sequenced on an Illumina HiSeq 4000.

### Chromatin-MS and WCE-MS

To evaluated the complex degradation efficiency, chromatin and whole cell extract (WCE) were subjected to mass spectrum analysis. For chromatin-MS, after auxin treatment, native cells were harvested for chromatin isolation, differently, for as much as possible to keep the proteins in chromatin, the urea buffer were replaced with 0.6% Triton X-100 to fractionate the chromatin. Isolated chromatin was directly dissolved and lysed using 1.5 × SDS-PAGE loading buffer, boiled for 10 min. For WCE-MS, cells were directly lysed using 1.5 × SDS-PAGE loading buffer, boiled for 10 min. 20 μg/lane protein was loaded for SDS-PAGE, and Coomassie Brilliant Blue stained gel samples were subjected to Orbitrap Fusion Lumos Tribrid Mass Spectrometer for label free quantitative analysis.

### TurboID IP-MS

For investigate the effects of the degradation of INO80, P400, SRCAP. The TurboID tag was knocked into the *C*-terminal of the Pol II largest subunit RPB1 in the INO80, P400, and SRCAP degron, respectively. To decrease the background, biotin in the 2i medium was depleted to a very low concentration using the streptavidin, which enough maintained the mESC growth, after treatment of Dox and auxin, 100 μM biotin was added into the medium for 30 min labeling, then washed cells 5 times with ice cold PBS. Finally, cells were harvested and directly lysed using 1 ml RIPA (strong) buffer for 10-cm dish cells. For enrichment of biotinylated proteins, 100 μl RIPA lysis buffer prewashed streptavidin magnetic beads (Invitrogen, M280) were added and incubated with clarified lysates with rotation at 4°C overnight. The beads were then washed twice with 1 ml of RIPA buffer, once with 1 ml 1 M KCl, once with 1 ml 0.1 M Na_2_CO_3_, once with 1 ml 2 M urea in 10 mM Tris·HCl (pH 8.0), and twice with 1 ml RIPA buffer [67]. The beads were subsequently eluted by boiled for 10 min in 2 × SDS-PAGE loading buffer supplement 2 mM biotin. Transferred the elute to new proteinase free tube and shipped for further processing and preparation for LC-MS/MS analysis.

### Chromatin MS, WCE MS, Turbo ID MS analyses

The peptides were mapped using the Proteome Discoverer software. The abundances for each protein (calculated as the summation of its associated and used peptide group abundances) were used for the differential analysis. Proteins with missing value in any sample were discarded. For Chromatin-MS, proteins annotated as non-nuclear or mitochondria proteins in UniProt database were also discarded. In order to perform differential analysis, the abundances for the remaining proteins were firstly normalized using TMM method from the R package EdgeR [68]. The normalized abundances were then analyzed using the R package DEP [69], which incorporates Limma as the method for differential analysis. Proteins with *P*-value < 0.05 and fold change > 1.5 were defined as significantly altered proteins.

For RPB1 TurboID-MS, proteins were annotated as participating in transcriptional initiation, elongation, termination and 3′ end processing processes according to the GO term (GO:0 006 367, GO:0 006 368, GO:0 006 369, GO:0 031 123, respectively) from MGI database. Proteins were annotated as histone acetyltransferases and demethylases according to the GO term (GO:0 004 402, GO:0 032 452, respectively) from MGI database. The interaction network of these proteins was taken from STRING database (confidence score > 0.9) and then visualized using Cytoscape software. Each protein was represented as a pie chart showing the log2 fold change under each of the three perturbed conditions.

### ChIP-seq, CUT&Tag, ATAC-seq analyses

Raw reads were firstly trimmed by Cutadapt [70] software and then mapped to mm10 and genome by Bowtie2 [71] software. Reads with spike-in were mapped to both mm10 and hg19 genome. PCR duplicated, multiple mapped and discordant reads were discarded for further analysis. Bam files were converted to bw format by deepTools [72] software using RPGC as the normalization method. Bam files with spike-in were normalized by the reads mapped to hg19 genome. Peaks were called by MACS2 [73] software for each replicate. The overlapped peaks were selected as the final peak set.

Typically, we use 5% of cells from different species as spike-in controls and perform standard normalization procedures, both with and without spike-ins, as the initial step in data processing. To ensure data accuracy and mitigate potential misinterpretations, we additionally performed independent qPCR validation and Western blot analyses to assess global changes in protein levels within chromatin fractions, where spike-in normalization is essential. These complementary methodologies provide robust validation of our experimental findings. It is crucial to highlight that normalization based on total library size assumes that overall signal levels remain comparable across samples. However, this assumption becomes invalid when the global efficiency of CUT&Tag reactions is affected—whether by experimental perturbations or technical variability. For instance, if a particular condition exhibits a significant reduction in total read counts, potentially due to decreased chromatin accessibility or altered antibody binding efficiency, the absence of spike-in normalization would fail to adequately correct for this global signal loss. Consequently, this could lead to an artificial inflation in the number of “upregulated” peaks, driven by relatively higher signals in localized regions rather than genuine biological changes. By incorporating spike-in normalization, we ensure a more accurate and reliable interpretation of the data, minimizing the risk of such artifacts.

The differential analysis was performed by DESeq2 [74] software using spike-in reads as size factors. TSS regions were defined as ± 2kb from transcriptional start sites. TES regions were defined as ± 2kb from transcriptional end sites and the sites that do not overlap with TSS regions. Regions with FDR < 0.05 were defined as significantly changed sites. The meta and heatmap plots were plotted using deepTools software. In order to perform quantile normalization for subsets of samples, the untreated samples were firstly divided into 10bp bins and then quantile normalized using the R package preprocessCore. The treated samples were re-scaled according to the fold change for each bin in the untreated samples compared with quantile normalized samples.

To enable a clearer understanding of how co-binding relationships relate to gene regulation, and to allow dissection of complex chromatin-remodeling dynamics, we define the co-bound or co-regulated sites or genes as follows: The “all” group includes promoters that overlap with peaks from all three complexes (INO80, P400, and SRCAP). “INO80 only” denotes promoters bound uniquely by INO80, without overlapping peaks from P400 or SRCAP. Similarly, “INO80 & P400” represents promoters co-occupied by both INO80 and P400, but not SRCAP. “INO80 & SRCAP” includes promoters bound by both INO80 and SRCAP only, while “other” includes promoters not fitting into any of these categories. These classifications were used to analyze changes in chromatin features (e.g. H2A.Z, BRD2) and transcription (e.g. Pol II occupancy and TT-seq signal) upon degron-mediated depletion of INO80 or P400.

### TT-seq and ChAR-seq analyses

Raw reads with spike-in were firstly trimmed by Cutadapt software and then mapped to mm10 and dm6 genome using Bowtie2 software. PCR duplicated, multiple mapped and discordant reads were discarded for further analysis. Bam files were converted to bw format by deepTools software normalized by the reads mapped to dm6 genome. Reads mapped to gene body regions (+300 bp to TES) of each gene were counted using featureCounts software. Genes with differentially expression (DEGs) were identified by DESeq2 software (FDR < 0.05, |log2FC| > 1). For TT-Seq upon the degradation of INO80 and P400, in order to include enough number of genes to define the direct target genes, we set the threshold of differentially expressed genes as FDR < 0.05 and |log2FC| > log_2_1.5.

The direct target genes were defined as genes met the following three criterions. (1) The binding strength of the remodeler at TSS region should reduce by at least 50% after the degradation of the remodeler. (2) The binding signal of RPB1 at TSS or TES region should significantly change (FDR < 0.05 identified by DESeq2) after the degradation of the remodeler. (3) The expression of the genes identified by TT-Seq should also significantly changed (defined as DEGs above) after the degradation of the remodeler.

In order to identify the factors enriched at the direct target genes, we gathered various types of features: (1) ChIP-seq of 169 factors. Around 1763 ChIP-seqs performed in mESCs were firstly downloaded from cistrome [75] database. Then a total of 169 data were carefully selected based on whether they were generated in V6.5 cells, whether they were perturbated and data quality. ChIP-seq signals of these factors at ± 500 bp around TSS site were used for further analysis. (2) ChIP-seq of 9 histone markers and CTCF. ChIP-Seq signals at ± 500 bp around TSS sites for H3K27ac, H3K9ac, H3K4me1, H3K4me2, H3K4me3, H3K79me2, H3K9me3, H3K27me3, and CTCF; ChIP-Seq signals at +500 to TES sites for H3K36me3; ChIP-seq signals at closest enhancers for H3K27ac, H3K9ac, H3K4me1, H3K4me2, and H3K4me3 were used for further analysis. (3) 12 core-promoter motifs. Core-promoter motifs were searched at ± 100 bp around TSS sites for each gene using MEME [76] software with “–max-stored-scores” set to 100000000. Each gene was assigned 12 motifs with the highest predicted score. The highest predicted score for each motif were used for further analysis. (4) Poly A sites. The number of poly A sites at various gene locus (terminal exon, other exon, downstream of terminal exon, antisense exon, antisense intron, antisense upstream of a gene and intergenic region) were downloaded from PolyASite [77] database and used for further analysis. (5) CpG. BioCAP-seq (GSM1064680) signals at ± 500 bp around TSS sites were used for further analysis. (6) Gene length, exon number and gene expression.

The gathered features were used to train a model to distinguish directed target genes with control genes randomly selected from all the active genes left to the same number of direct target genes. Firstly, near-zero features were discarded. Features with correlations larger than 0.7 were also filtered to avoid multicollinearity. Then Z-score for each feature was used to train an elastic net model using LogisticRegression from the scikit-learn package. The hyper-parameters were searched using GridSearchCV using 3-fold cross validation and roc_auc score as the scoring method. Finally, the coefficient for each feature from the best model were used to access the contribution of the feature in distinguishing the genes in the two categories.

### RNA-seq analysis

Raw reads with spike-in were firstly trimmed by Cutadapt software and then mapped to mm10 and dm6 genome using STAR [78] software. PCR duplicates, reads that were not primarily mapped were discarded for further analysis. Bam files were converted to bw format by deepTools software normalized by the reads mapped to dm6 genome. Reads mapped to each gene were counted using featureCounts [79] software. The changes of splicing events were evaluated by rMATS [80] software, where PSI values were calculated for each genes and genes with FDR < 0.05 were defined as splicing changes. GO enrichment analysis were identified by the R package clusterProfiler. The number of each sets of DEGs under the three different perturbations in a GO term was shown as the proportion in a pie plot. The proportion of DEGs mapped on a GO term were adjusted for duplicated genes and gene sizes in a same way as ClueGO [81] did. The location of each pie was displayed using the *P*-value and gene ratio of the enrichment results for DEGs sets in that term with the largest proportion. To identified the most active transcript for each gene, we firstly count the reads that mapped to each transcript using StringTie [82] software. Then transcripts with the largest TPM were kept. Longer transcripts were selected if the TPM value of all the transcripts are the same.

The splicing analysis were performed using rMATs software [83]. The software can automatically detect and analyze alternative all major types of splicing events from polyA RNA-seq data, including skipped exon, alternative 5′ splice site, alternative 3′ splice site, mutually exclusive exons and retained intron, by evaluating the reads mapped to exons and splice junctions. It also uses a statistical model to determine the changes in the isoform ratio of a gene under two conditions and provides *P*-value and FDR value for the comparison. In Supplementary Fig. S3E, we used rMATs for splicing analysis, merge the splicing events together and found changes of isoform ratio for subsets of genes.

### ChIP-MS analysis

The peptides were mapped using Proteome Discoverer software. The PSM value for each protein were used for the analysis. Proteins annotated as non-nuclear or mitochondria proteins or high abundance heat shock proteins in UniProt database were also discarded. Proteins with missing value were imputed as the smallest value in the library. The enrichment score was defined as log2 (mean(IP)+1) /(input+1), and proteins with enrichment score larger than 1 were used for GO enrichment analysis.

### Statistical analysis

For RNA-Seq, ChAR-Seq, TT-Seq, ChIP-Seq, CUT&Tag, ChIP-qPCR, and RT-PCR experiments, two biological replicates were performed. For ChIP-MS, TurboID-MS and WCE-MS expreiments, three biological replicates were performed. Statistical analysis was performed using Wilcoxon test or *t*-test according to the figure legends. Almost all the described data-processing and analyzing steps were performed in R, GraphPad and Microsoft Excel. Custom code used in this study is available upon request.

## Results

### The validation of INO80/SWR remodelers degron in mESCs

**Figure 1.**
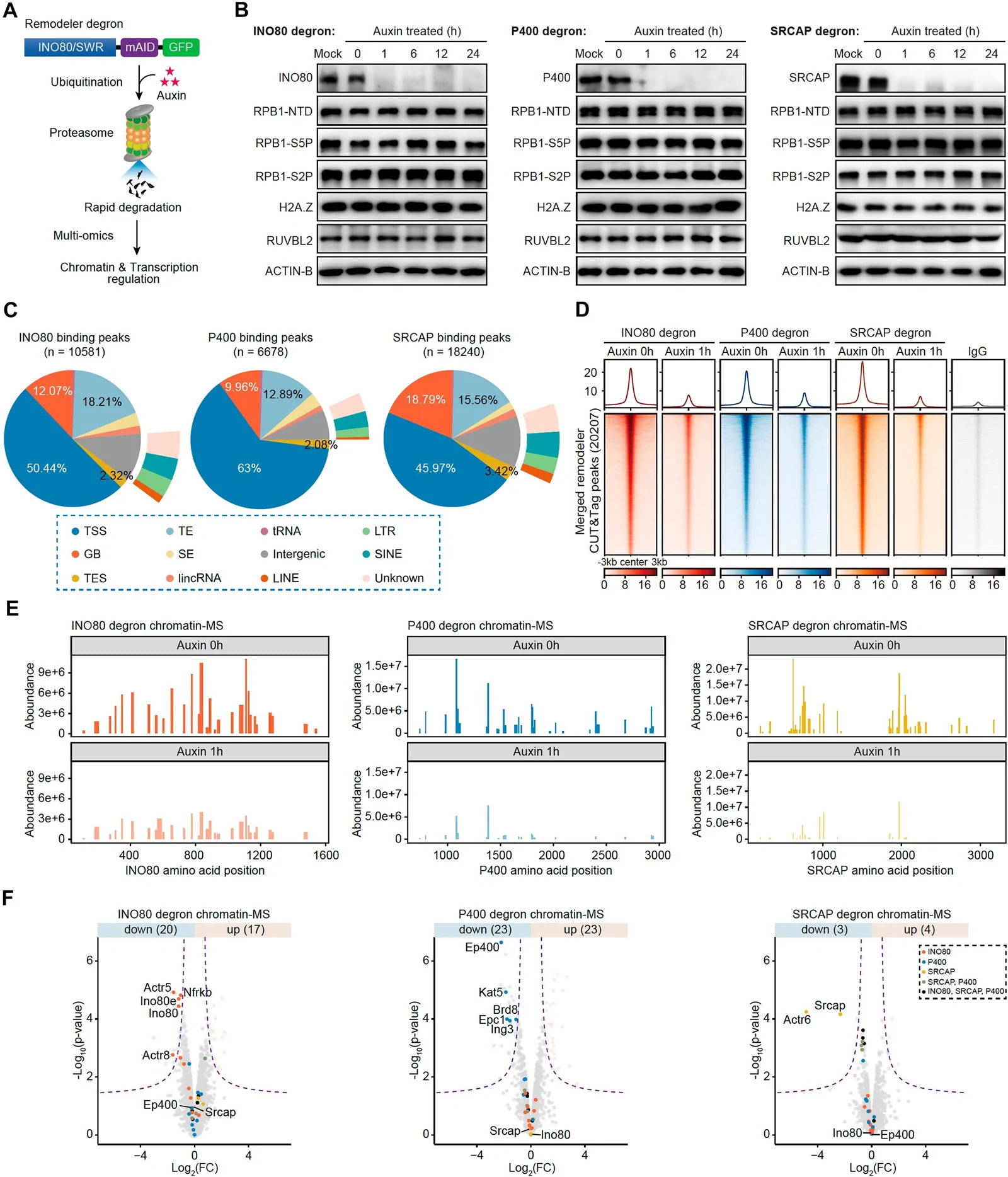
Specific degradation of INO80, P400, and SRCAP complexes in mESCs. (**A**). Diagram illustrating the *C*-terminal degron of the largest subunit of INO80, P400, and SRCAP chromatin remodelers, enabling their targeted protein degradation upon auxin addition. (**B**). Western blot analysis to confirm the degradation of INO80, P400, and SRCAP, with different phosphorylation states of Pol II and other proteins serving as controls. (**C**). Genomic distribution of INO80, P400, and SRCAP CUT&Tag peaks, depicting their binding sites across the genome. GB, TE, SE indicate the gene body, typical enhancer and super-enhancer, respectively. (**D**). Heatmap displaying the chromatin binding signal of INO80, P400, and SRCAP before and after their degradation. Signals were centered around ± 3 kb of merged peaks of INO80, P400, and SRCAP. (**E**). Quantification of the abundance changes of mAID-fused proteins in the chromatin fractions. Each bar at different positions represents the abundance of a peptide group. (**F**). Volcano plot showing the proteins with altered abundance in chromatin-mass spectrometry upon INO80, P400 and SRCAP degradation. The decrease in Actr6 may be attributed to its close interactions with SRCAP in the chromatin.

Previously, we employed auxin-inducible degron to degrade the shared subunits of INO80/SWR remodelers and found that RUVBL1/2 directly regulates Pol II clustering at active gene promoters, but we observed minimal effects on chromatin occupancy of P400 and INO80 [28]. We used the same technique to acutely degrade the largest subunits of INO80, P400/TIP60, and SRCAP complexes in mESCs (Fig. 1A). We homogenously inserted the degron-GFP tag into the *C*-terminal of the target genes (*Ino80*, *P400* and *Srcap*) using CRISPR/Cas9 gene editing. This tagging did not negatively affect the targeted genes, as previously reported for RNA polymerase subunits [84]. Western blotting analyses indicated that 1 h auxin treatment caused degradation of the targeted proteins with little impact on the protein levels of different phosphorylation states of Pol II, H2A.Z, and RUVBL2 (Fig. 1B).

CUT&Tag experiments were conducted using the degron cells due to the limited effectiveness of ChIP-seq experiments in capturing SRCAP interactions with chromatin, indicating its more dynamic association. INO80, P400, and SRCAP CUT&Tag with GFP antibodies identified 10 581, 6678, and 18 240 peaks, respectively (Supplementary Table S1). They preferentially bind to gene regions and typical enhancers and co-occupy around 50% across the genome and consistent with previously reported binding sites (Fig. 1C, Supplementary Fig. S1A and B) [85, 86]. The motifs of their peaks were identified (Supplementary Fig. S1C). The top enriched motifs for all three remodelers are predominantly associated with zinc finger transcription factors, particularly those recognizing GC-rich sequences. While all three remodelers are associated with promoter-proximal binding and GC-rich motifs, they may interact with distinct sets of transcription factors or cofactors that could contribute to their functional specificity. For example, INO80 shows a strong preference for KLF and SP family members, while P400 and SRCAP exhibit unique enrichments for factors such as NRF1, SREBF2, and SMAD4, respectively. These differences could reflect divergent roles in regulating specific transcriptional programs or chromatin states. Their chromatin binding strengths were weakly correlated with the mRNA gene expression level (Supplementary Fig. S1D). The auxin treatment dramatically decreased their chromatin binding (Fig. 1D, Supplementary Fig. S1E and F), indicating these CUT&Tag binding signals were specific.

Quantitative mass spectrometry analyses of chromatin fractions were performed after INO80, P400, and SRCAP depletion (Supplementary Table S2). The peptide densities of INO80, P400, and SRCAP were uniformly decreased (Fig. 1E), indicating the degradation of these proteins. Further analyses showed that peptide densities of the proteins spatially close to the degron protein were relatively more reduced than spatially distant ones based on the structures of INO80/SWR remodelers (Supplementary Fig. S1G) [58, 59, 87–92]. Moreover, we observed minor effects on specific components of other remodelers, some of which showed decreasing trends that did not meet our statistical significance threshold (Fig. 1F). In contrast, similar analyses of WCE consistently showed decreased protein levels of INO80, P400 and SRCAP, while most other subunits did not exhibit significant changes (Supplementary Fig. S1H). Only a limited number of other proteins exhibited changes, but these changes were not enriched in specific functional pathways. Together, these findings provide a comprehensive evaluation of auxin-inducible degron for INO80/SWR remodelers, suggesting their degradations are specific in mESCs and offering valuable systems for investigations of INO80/SWR remodelers’ functions.

### The loss of INO80/SWR remodelers lead to diverse defects of Pol II transcription

**Figure 2.**
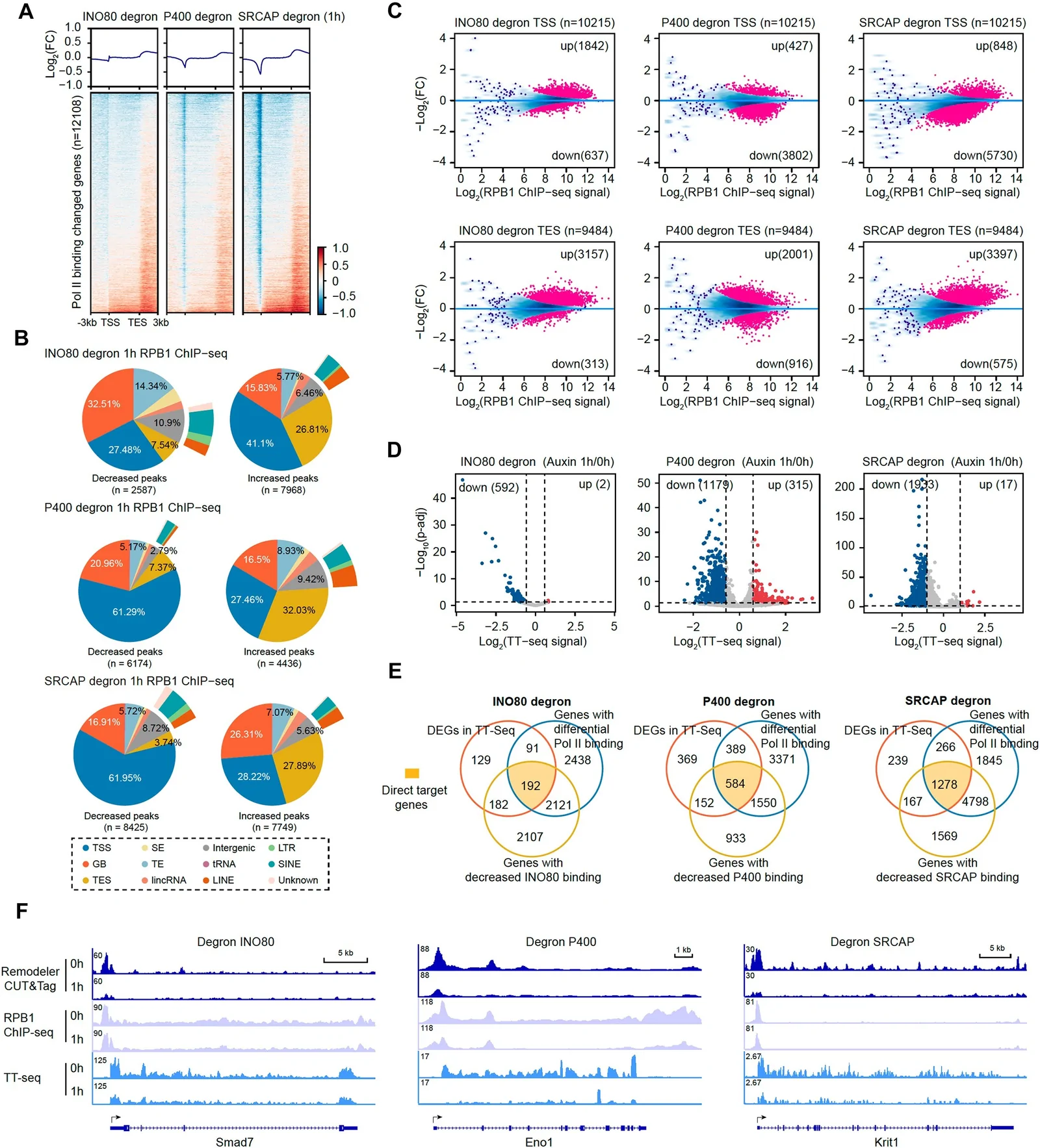
Direct target genes of INO80/SWR remodelers were identified in mESCs. (**A**). Heatmap showing the log2 fold change of Pol II ChIP-Seq signal at all the active genes. (**B**). Genomic distribution of the peaks with increased or decreased Pol II signal before and after INO80, P400, and SRCAP degradation for 1 h, respectively. The differentially binding peaks were identified by DESeq2 software (FDR < 0.05). (**C**). MA-plots for the changes of Pol II signals at TSS regions and TES regions upon INO80, P400, and SRCAP degradation for 1 h. Regions with increased or decreased Pol II binding were identified by DESeq2 software (FDR < 0.05) and marked red. (**D**). Volcano plots showing the differentially expressed genes identified by TT-seq experiment after 1 h of INO80, P400, and SRCAP degradation. The differentially expressed genes were identified using DESeq2 software (FDR < 0.05, absolute fold change >1.5 for INO80 and P400, absolute fold change > 2 for SRCAP). (**E**). Venn diagrams showing the definition of direct target genes by overlapping the differentially expressed genes from TT-seq, the differentially bound genes identified by Pol II, and the genes showing more than a 50% decrease in remodeler subunit binding following 1 h degradation of INO80, P400, and SRCAP. (**F**). Genome browser tracks showing remodeler (INO80, P400, SRCAP), RPB1 (Pol II), TT-seq (newly transcribed RNA) signals at Smad7 (INO80-regulated), Eno1 (P400-regulated), and Krit1 (SRCAP-regulated) loci in control (top) and 1-h degradation conditions (bottom).

In order to investigate the regulation of INO80/SWR remodelers on Pol II transcription, we performed Pol II ChIP-seq after degrading INO80, P400, or SRCAP for 1 h (Fig. 2A, Supplementary Table S3). Genome distribution analyses of increased and decreased Pol II peaks were conducted, revealing that these peaks are mostly affected in gene regions such as transcription start sites (TSS), gene body (GB), and transcription termination sites (TES) (Fig. 2B). Differential analyses of Pol II ChIP-seq signals were also performed and displayed with MA plots. The results showed that INO80 depletion preferentially increased Pol II occupancy at the TSS and TES of many genes, reminiscent of previous findings that suggested INO80 depletion led to increased cryptic transcription [85, 93]. P400 and SRCAP degradations caused a preferential decrease in Pol II at the TSS and an increase in Pol II at the TES (Fig. 2C). Notably, some of these genes were subsequently validated through ChIP-qPCR experiments (Supplementary Fig. S2A and B). We also conducted Pol II ChIP-seq following 24 h of auxin treatment and observed a decrease in the Pol II ChIP-seq signal for P400 and SRCAP. In contrast, the Pol II ChIP-seq signal for INO80 did not exhibit obvious changes (Supplementary Fig. S2C).

We conducted transient transcriptome sequencing (TT-seq) to observe which genes had their nascent RNA production affected following the degradation of the three INO80/SWR remodelers. Our study revealed that a decrease in nascent RNA was the predominant trend across all three remodelers (Fig. 2D). To illustrate the overlapping genes among the differentially expressed genes from TT-seq, the differentially affected genes identified by Pol II ChIP-seq, and the genes showing more than a 50% decrease in remodeler binding after depletion, we created a Venn diagram. We defined these overlapping genes as the direct target genes of the remodelers and focused our subsequent analysis on the changes within this gene set for the rest of the study (Fig. 2E, Supplementary Table S5). We conducted Gene Ontology (GO) analyses on the direct target genes identified in our study and found that these genes share common associations with ribosome biogenesis and RNA processing, while also exhibiting distinct functional enrichments. Specifically, the direct target genes of INO80 are predominantly enriched in processes related to chromatin organization. For P400, the direct target genes are primarily enriched in processes associated with DNA replication and chromatin segregation. SRCAP’s direct target genes show significant enrichment in processes linked to DNA repair and cilium organization (Supplementary Fig. S2D). These findings suggest that while all three remodelers contribute to RNA processing, their specific roles diverge in ways that might influence distinct aspects of development and disease.

We have conducted a detailed investigation into the direct target genes of INO80, P400, and SRCAP following their degradation (Fig. 2F). Specifically, we selected Smad7 (regulated by INO80 and is a key negative regulator of TGF-β signaling, controlling cell differentiation and immune responses.) [94, 95], Eno1 (regulated by P400 and is a glycolytic enzyme essential for metabolism and implicated in cancer progression) [96], and Krit1 (regulated by SRCAP and stabilizes endothelial cell junctions and is linked to vascular integrity and cerebral cavernous malformations) [97, 98], and analyzed their transcriptional dynamics using multiple assays, including Pol II ChIP-seq and TT-seq. Our results consistently show a reduction in Pol II ChIP-seq and TT-seq signals after degradation, indicating a significant decrease in transcriptional activity at these loci. These findings imply that acute depletion of these remodelers disrupts transcriptional output at functionally important targets, potentially altering downstream pathways (e.g. TGF-β suppression, metabolic flux, or vascular stability).

### The H2A.Z occupancy and chromatin accessibility are affected after the acute depletion of INO80/SWR remodelers

**Figure 3.**
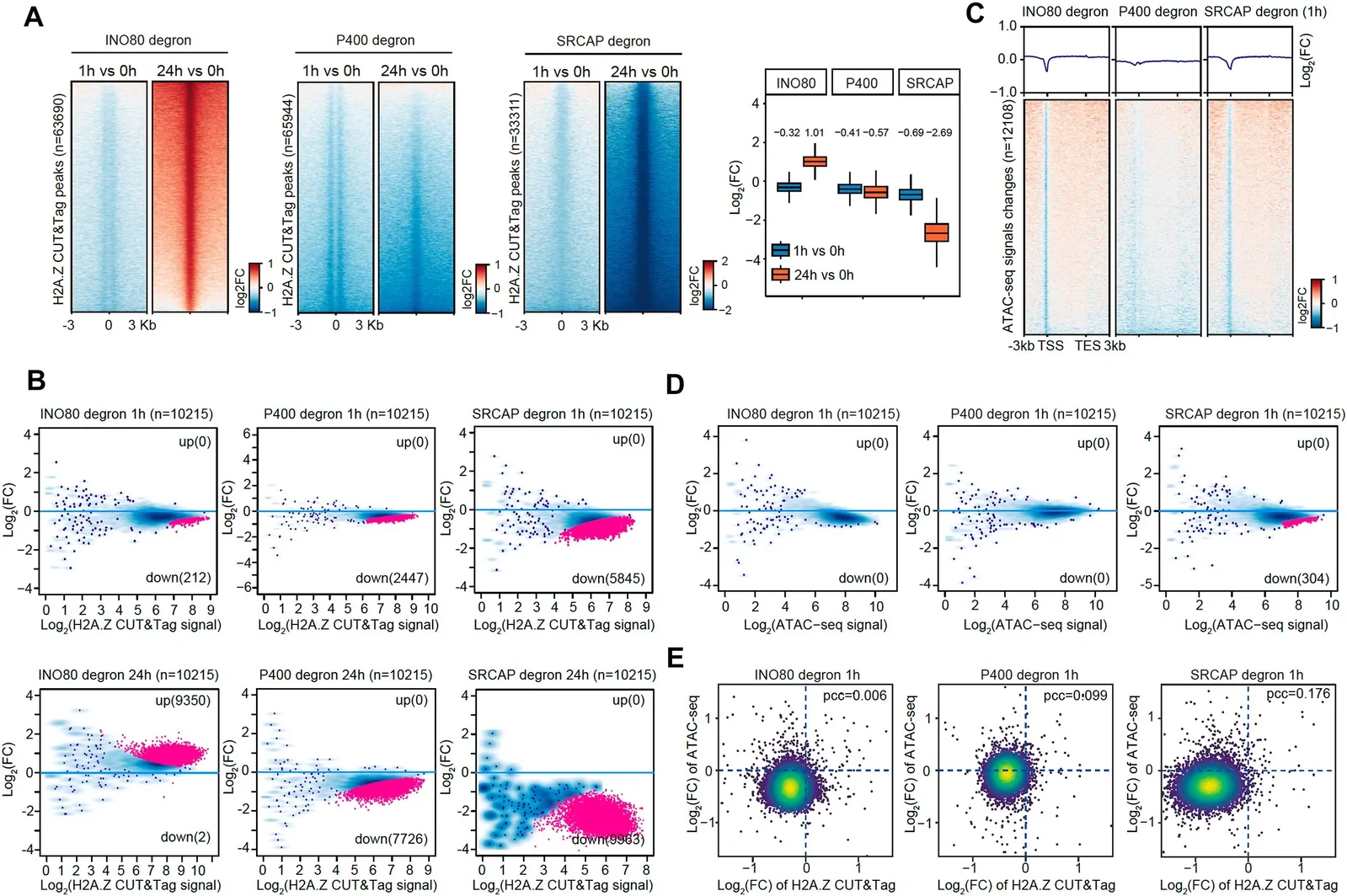
Depletion of INO80/SWR remodelers for 1 and 24 h differentially affects H2A.Z occupancy. (**A**). Heatmap showing log2 fold change of H2A.Z signal at merged peaks of INO80, P400, and SRCAP following 1 and 24 h degradation of INO80, P400, and SRCAP. The box plots illustrate the quantification of these changes. (**B**). MA-plots for the changes of H2A.Z CUT&Tag signals at TSS regions after 1 and 24 h degradation of INO80, P400, and SRCAP. Regions with increased or decreased H2A.Z binding were identified by DESeq2 software (FDR < 0.05) and marked red. (**C**). Heatmap showing the log2 fold change of ATAC-seq signals at all the active genes upon INO80, P400 and SRCAP degradation. (**D**). MA-plots for the changes of ATAC-seq signals at TSS regions upon INO80, P400 and SRCAP degradation. Regions with increased or decreased chromatin accessibility were identified by DESeq2 software (FDR < 0.05) and marked red. (**E**). Dot plots showing the correlation of the changes of H2A.Z CUT&Tag and ATAC-seq upon the degradation of INO80, P400, and SRCAP.

Nucleosomes act as transcriptional barriers, and the incorporation of H2A.Z can modulate Pol II transcription [18, 20]. Previous biochemical evidence has shown that the INO80/SWR remodelers regulate H2A.Z dynamics [33–37], but their immediate effects in mammalian cells have not been clear. We performed H2A.Z CUT&Tag and ATAC-seq after acute depletion of INO80, P400, and SRCAP for 1 h. The results showed that INO80 depletion had minimal impact on H2A.Z occupancy, while P400 degradation led to decreased (n = 2 447) H2A.Z occupancy and SRCAP degradation resulted in decreased occupancy at 5 845 TSSs of genes (Fig. 3A and B), and further validated with H2A.Z CUT&Tag qPCR and chromatin fraction Western blot analyses (Supplementary Fig. S2E and F). The MA-plot and meta-gene analyses indicated subtle alterations in chromatin accessibility after 1 h of INO80/SWR remodelers’ depletion (Fig. 3C and D). We performed the correlation analyses of H2A.Z occupancy changes with chromatin structures assayed by ATAC-seq. The results suggested that upon remodeler degradation, the observed changes in H2A.Z occupancy align with alterations in chromatin accessibility (Fig. 3E), indicating a coordinated regulation of H2A.Z incorporation and chromatin structure.

We further analyzed the data by categorizing genes based on their binding profiles: those bound by all three proteins (INO80, P400, and SRCAP), those bound by only one (e.g. only INO80), or those bound by specific pairs (e.g. only INO80 and P400, or only INO80 and SRCAP). Our analysis focused on active genes revealed that H2A.Z occupancy changes are largely independent of whether the gene is bound by one, two, or all three remodelers. However, H2A.Z levels remained unchanged only in genes that were not affected and not bound by INO80/SWR remodelers (indicated as the gray portion in the figure) (Supplementary Fig. S2G). We also performed correlation analyses of changes in nascent transcription assayed by TT-seq after remodeler degradation with nascent transcription assayed by NET-seq after H2A.Z degradation from a previous study [20]. The results showed little correlation (Supplementary Fig. S2H), suggesting that these remodelers’ roles in transcription may not be directly mediated through H2A.Z or may involve additional regulatory layers. We also conducted CUT&Tag experiments for H2A.Z after long-term (24 h) degradation of INO80/SWR remodelers. Our results are consistent with previous studies showing that prolonged degradation of P400 and SRCAP leads to reduced H2A.Z incorporation in chromatin (Fig. 3A and B) [33, 34, 37]. However, the INO80 degradation for a longer time caused the increased H2A.Z occupancy (Fig. 3A and B). These results suggest that long time depletion of the INO80/SWR remodelers may induce a complex impact on H2A.Z dynamics in cells.

### The INO80, P400/TIP60, and SRCAP remodelers are responsible for the expression of different genes

In order to gain more understanding about INO80/SWR remodelers-mediated gene expression, we conducted chromatin-associated RNA sequencing (ChAR-seq) and total Poly (A) RNA sequencing (RNA-seq) analyses after depleting these remodelers. Correlation analyses of the ChAR-seq and RNA-seq data showed similar gene expression among three degron cells under untreated condition (Supplementary Fig. S3A and B), indicating that the degron tag did not have adverse effects on the cells as reported previously [56, 84]. The results from 1 h of target protein depletion ChAR-seq analyses indicated that these remodelers significantly affected the expression of a subset of genes, and the RNA-seq analyses after 24 h depletion also led to dysregulations of subsets of genes, specific to different remodelers (Supplementary Fig. S3C and D). Changes in Pol II ChIP-seq were global, but RNA expression changes were significant for subsets of genes, suggesting that these remodelers may also be involved in co-transcriptional or post-transcriptional RNA processing, or that the discrepancies may arise from differences in experimental quantification. As expected, splicing defects were observed after the depletion of these remodelers (Supplementary Fig. S3E); however, the potential mechanisms remain unclear.

GO analysis on ChAR-seq data following 1 h degradation of INO80, P400 and SRCAP, which enriched for terms related to RNA processing and metabolism (Supplementary Fig. S3F). In contrast, Gene ontology analyses of RNA-seq data from genes with altered expression after 24 h of degradation revealed that INO80 is primarily required for gene expression related to metabolic processes, P400 is essential for developmental genes, and genes affected by SRCAP are enriched in RNA modification and cilium assembly (Supplementary Fig. S3G). We then conducted RNA-seq after 1 h of remodeler degradation. The results showed minimal changes in gene expression (Supplementary Fig. S3H). Consequently, longer periods of remodeler depletion may lead to secondary effects, such as the global impact on transcription may affect mRNA degradation pathway [99].

### INO80/SWR remodelers regulate Pol II transcription via BRD2

**Figure 4.**
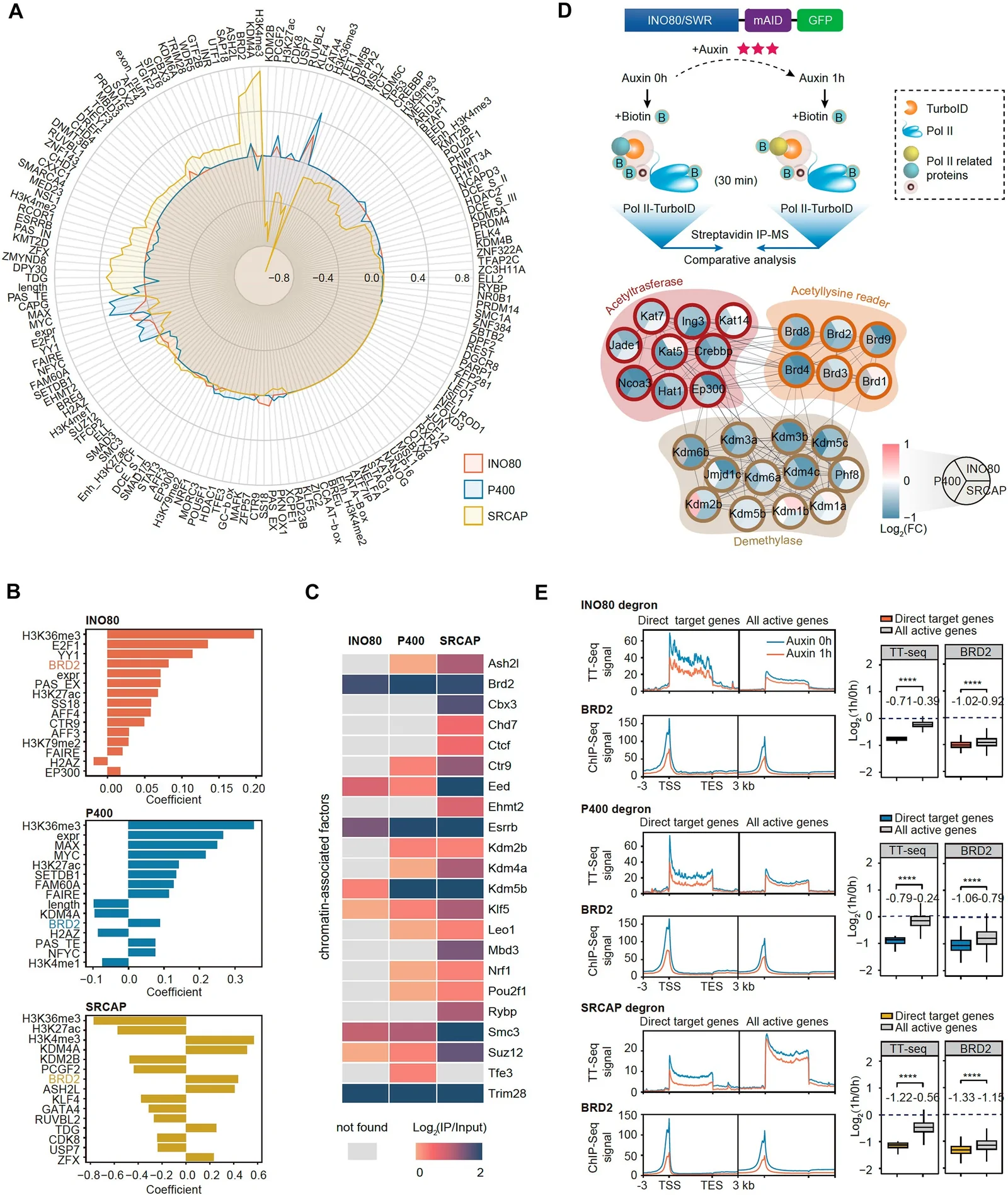
INO80/SWR remodelers regulate Pol II transcription via H2A.Zac reader BRD2. (**A**). Radar plot showing the coefficient from GLM model (see method for detail). (**B**). The bar chart displaying the coefficients of the top 15 factors with the largest absolute coefficient from the GLM model. (**C**). Some factors selected from the GLM model are enriched in ChIP-MS of INO80, P400, and SRCAP. (**D**). Top: A schematic diagram illustrating the Pol II-TurboID system in mESCs upon INO80, P400, and SRCAP degradation. Bottom: Changes in the interaction of acetyllysine reader proteins, acetyltransferases, and demethylases with Pol II identified by Turbo-ID MS upon INO80, P400, or SRCAP degradation. The color bar indicates log2 fold change. (**E**). Meta-analysis showing the average values of TT-seq and BRD2 ChIP-Seq signals, for direct target genes and all active genes before and after 1 h of INO80, P400, and SRCAP degradation. Blue indicates pre-degradation, and red indicates post-degradation. Box plots display the log2 fold changes of TT-seq and BRD2 ChIP-Seq signals for direct target genes (*n* = 192, 584, 1 278 for INO80, P400, and SRCAP, respectively) and all genes (*n* = 12 108). Red indicates direct target genes, and grey indicates all genes. Statistical analysis was determined using the Wilcoxon test. * *P*< 0.05, ** *P*< 0.01, *** *P*< 0.001, **** *P*< 0.0001. The log2 fold change values were displayed above the box plot.

To gain further mechanistic insights, we analyzed publicly available ChIP-seq data (Supplementary Table S6), gene/enhancer features, and core-promoter motifs for INO80/SWR remodelers direct target genes. We trained the machine learning model to see if the selected features can distinguish between the direct target genes and non-direct target genes. We conducted generalized linear model (GLM) machine learning to identify features using the direct target genes (Fig. 4A, Supplementary Fig. S4A). In the GLM, larger coefficients indicate a clearer distinction between direct target genes and non-direct target genes. We present the top 15 ranked factors, and found that histone modifications and related enzymes consistently ranked highly across all three groups. For instance, H3K36me3, BRD2, H3K27ac, H2A.Z, and EP300 are associated with INO80, while H3K36me3, H3K27ac, KDMA4A, BRD2, and H2A.Z are linked to P400. Determinants affecting gene regulation by SRCAP also include H3K36me3, H3K27ac, H3K4me3, KDMA4A, KDMA2B, and BRD2 (Fig. 4B). This suggests a strong correlation between the transcriptional changes induced by the three INO80/SWR remodelers and histone modifications. Based on our INO80/SWR remodelers ChIP-MS data, we found that BRD2 preferentially interacts with these remodelers (Fig. 4C, Supplementary Fig. S4B–D). Furthermore, the direct target genes of INO80/SWR remodelers significantly overlap with the genes whose transcription is affected by BRD2 depletion (Supplementary Fig. S4E). It is important to note that BRD2 depletion affected more than 5 000 genes [107], whereas the depletion of INO80/SWR remodelers had a smaller impact on the transcriptome, with only about 50% overlap with BRD2 target genes. This suggests that there may be alternative explanations for the roles of INO80/SWR remodelers in transcription, apart from their association with BRD2.

We next used proximal protein labeling to identify proteins associated with Pol II after INO80/SWR remodelers’ depletion and performed mass spectrometry analyses. TurboID, a widely used biotin ligase [67, 100], was knocked into the *C*-terminal of the largest subunit of Pol II (*Rpb1*) in our remodeler degron cells, allowing for covalent labeling of proximal proteins with biotin in living cells (Fig. 4D, Supplementary Fig. S5A–C) [67]. Biotin-labeled proteins were purified by streptavidin pull-down, and differential analyses were performed with TurboID-MS before and after auxin treatment, revealing hundreds of proteins associated with Pol II that were affected (Supplementary Fig. S5D and E). GO analyses indicated that mRNA splicing, chromatin organization and 3′end processing factors were affected after INO80, P400, and SRCAP depletion (Supplementary Fig. S5F). Comparative analysis of RNA-seq and TurboID-MS datasets revealed minimal overlap, suggesting that changes in protein-proximity interactions with Pol II do not necessarily correlate with observable transcriptional changes (Supplementary Fig. S5G). Analysis of protein complexes from the STRING database showed that the interactions of histone acetyltransferases, acetyl lysine reader and integrator complex with Pol II are preferentially decreased after INO80/SWR remodelers’ depletion (Fig. 4D, Supplementary Fig. S5D). After the degradation of INO80/SWR remodelers, INTS5 CUT&Tag was subsequently performed, revealing more pronounced alterations in chromatin binding at TSS compared to TES regions (Supplementary Fig. S5H). A small percentage INTS5 affected genes overlapped with Pol II affected genes after the depletion of INO80 and SRCAP, and the changes in chromatin binding can be further validated through qPCR. In constrict, the P400 direct target genes show a large percentage overlap with the INTS5 decreased genes following P400 depletion (Supplementary Fig. S5I), suggesting that P400-mediated acetylation may play a role in integrator-associated transcription regulation. However, the relationship between the chromatin remodeling and INTS5 changes is not well understood. The degradation of INO80/SWR remodelers for 1 h consistently led to a decrease in BRD2 chromatin binding at direct target genes. We also observed that while all active genes exhibited a similar trend, the changes were less significant compared to those of the direct targets (Fig. 4E).

### The regulation of Pol II transcription by INO80/SWR remodelers is associated with certain chromatin landscape, such as H2A.Z acetylation, H3.3, and the binding of other INO80/SWR remodelers

**Figure 5.**
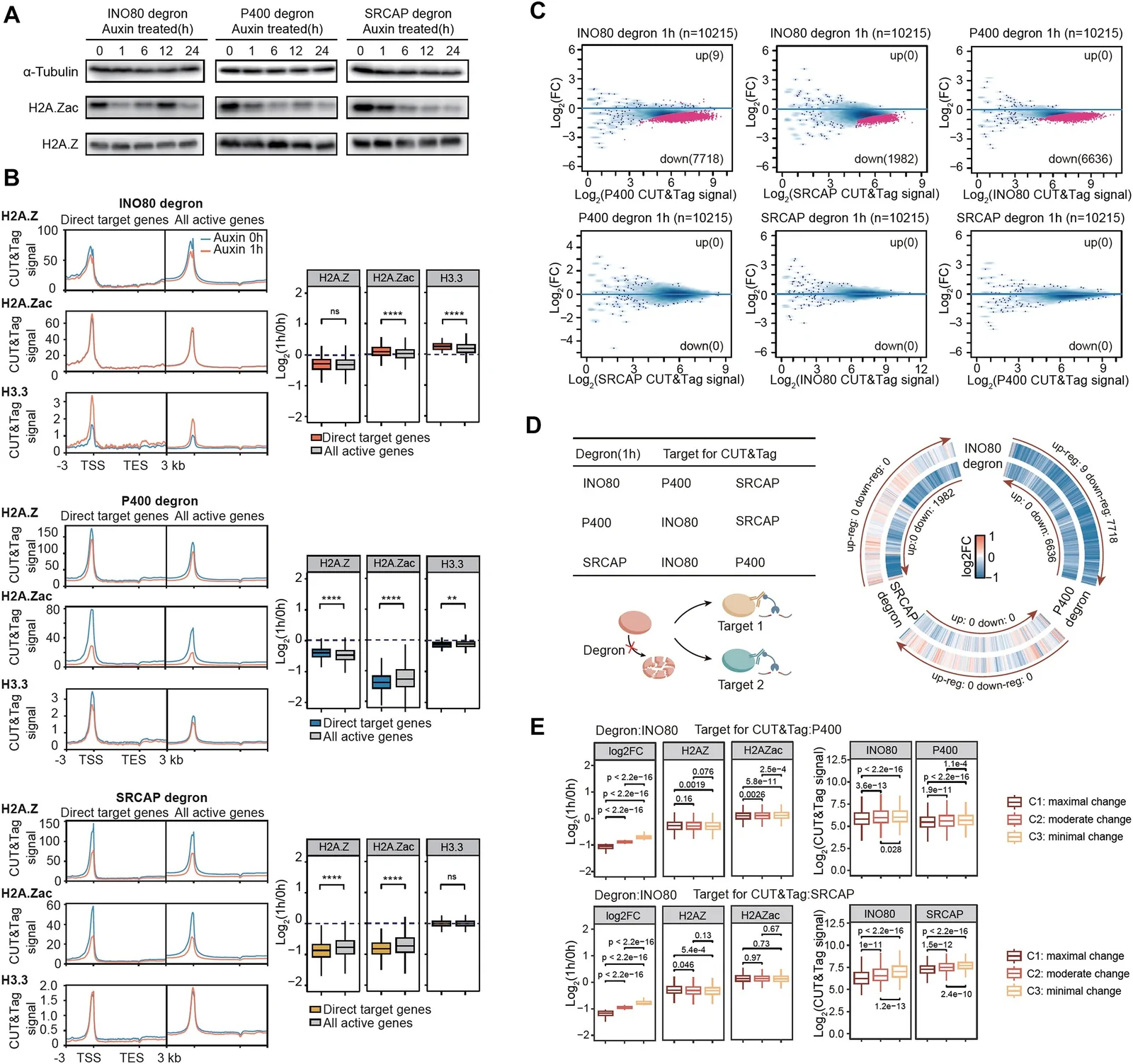
P400 and SRCAP help maintain H2A.Zac chromatin occupancy. INO80 appears to utilize an H2A.Zac-independent mechanism to regulate BRD2 and regulate the occupancy of P400 and SRCAP. (**A**). Western blot showing total H2A.Z acetylation levels at different time points of INO80, P400, and SRCAP degradation. Note: The Western blot results have been repeated three times, consistently showing a similar trend. (**B**). Meta-analysis showing the average values of H2A.Z, H2A.Z acetylation, and H3.3 CUT&Tag signals, for direct target genes and all active genes before and after 1 h of INO80, P400, and SRCAP degradation. Blue indicates pre-degradation, and red indicates post-degradation. Box plots display the log2 fold changes of H2A.Z, H2A.Z acetylation, and H3.3 CUT&Tag signals for direct target genes and all genes. Direct target genes are shown in different colours (red, blue, or yellow, depending on the experimental condition), whereas all active genes are shown in grey. Statistical analysis was determined using the Wilcoxon test. * *P*< 0.05, ** *P*< 0.01, *** *P*< 0.001, **** *P*< 0.0001. (**C**). MA-plots illustrating the log2 fold changes following the degradation of one INO80/SWR protein, as identified by CUT&Tag analysis of the remaining two proteins. The red dots indicate the genes with significantly changed signals identified by DESeq2 (FDR < 0.05). (**D**). Left: Schematic representation of the cross-regulation experiment. CUT&Tag assays were performed for the other two remodelers after degrading one remodeler. Right: Heatmap showing cross-regulation experimental results, with arrows originating from the degradation target and pointing to the target observed for changes. The color bar indicates log2 fold change. The starting point of each arrow represents the degraded protein, while the endpoint of the arrow indicates the protein subjected to CUT&Tag sequencing. The heatmap colors reflect the changes in sequencing signals across all active genes, providing a visual representation of the impact of degrading one remodeler on the occupancy of the others. (**E**). Left three boxplots showing the log2 fold change of P400 or SRCAP, H2A.Z, and H2A.Zac CUT&Tag signal upon the degradation of INO80 at three gene clusters. Right two boxplots showing the CUT&Tag signal of INO80, P400, or SRCAP at three gene clusters. The genes are classified into maximal (*n* = 2573 and 661 for P400 and SRCAP target respectively), moderate (*n* = 2572 and 660 for P400 and SRCAP target, respectively) and minimal (*n* = 2573 and 661 for P400 and SRCAP target, respectively) changes according to the log2 fold change of P400 or SRCAP CUT&Tag signals upon the degradation of INO80. Statistical analysis was determined using the Wilcoxon test.

We performed Western blot experiments at different degradation time points to observe the changes of H2A.Z acetylation within the cells. The results indicate that during the degradation of P400 and SRCAP from 1 to 24 h, H2A.Z acetylation levels gradually decreased. In contrast, the degradation of INO80 over the same period exhibited an initial trend of declining, followed by recovery, and then a subsequent decrease (Fig. 5A). To further validate the changes in H2A.Z acetylation at the transcription start site (TSS), we conducted CUT&Tag experiments. After the degradation of P400 and SRCAP, H2A.Z acetylation levels significantly decreased (Fig. 5B), with these findings further validated by qPCR (Supplementary Fig. S6A). Our results revealed that total H2A.Z levels remained unchanged in Western blot analyses, while CUT&Tag analyses showed a decrease in H2A.Z occupancy for INO80, SRCAP and P400. The inconsistency between these results could be explained by their different detection scopes while Western blot analyzes total cellular H2A.Z acetylation levels from whole-cell lysates, CUT&Tag specifically detects chromatin-associated H2A.Z modifications. The causal relationship between the reduction in H2A.Z chromatin occupancy and the observed decrease in H2A.Zac chromatin occupancy is not clear. Pol II has been identified as the primary factor facilitating the turnover of H2A.Z at the + 1 nucleosome; therefore, we believe that the effects of H2A.Zac are independent of changes in Pol II.

Our Western blot signals for H2A.Zac also decreased. Therefore, we conclude that the transcriptional defects associated with P400 and SRCAP are likely due to the decreased levels of H2A.Zac. The meta-analysis results revealed that after INO80 degradation, the binding of H2A.Z acetylation on chromatin remained unchanged, which is inconsistent with the Western blot results. This suggests that INO80 may also regulate the process of H2A.Z acetylation outside of chromatin, indicating a more complex mechanism involving interactions with other intracellular factors or changes for the dynamics of H2A.Zac. Based on previous reports highlighting the functional links between H2A.Z and H3.3 [101, 102]), we further examined the occupancy of the histone variant H3.3 and found that the degradation of INO80 led to a significant increase in H3.3 occupancy, while the depletion of P400 and SRCAP did not show any noticeable effects (Fig. 5B, Supplementary Fig. S6C). These results suggest that INO80/SWR remodelers differentially affect the dynamics of H2A.Z acetylation and H3.3, underlying their respective roles in the transcriptional process. However, the mechanisms explaining the changes in H3.3 are still unclear.

To explore the potential cross-regulations among different INO80/SWR remodelers, we degraded each of the three remodelers and performed CUT&Tag experiments for the remaining two. Our results revealed that the degradation of INO80 led to decreased chromatin occupancy of both SRCAP and P400, while the depletion of P400 resulted in reduced chromatin occupancy of INO80 (Fig. 5C and D). These phenomena can be further validated using qPCR (Supplementary Fig. S6D). Correlation analyses indicated that the decrease in SRCAP levels is associated with the binding strength of INO80, suggesting that INO80 may facilitate the chromatin recruitment of SRCAP (Fig. 5E, Supplementary Fig. S7A). To distinguish direct effects from rapid compensatory effects involving redistribution of these chromatin remodeling ATPases, we conducted additional analyses to explore their cross-regulation (Supplementary Fig. S7B): (1) Depletion of INO80 results in reduced chromatin binding of both P400 and SRCAP. However, the changes observed in H2A.Z, H2A.Zac, BRD2, Pol II, and TT-seq signals following INO80 degradation do not correlate with the reduced presence of P400 or SRCAP at gene promoters after INO80 depletion. This suggests that the downstream effects on these factors are primarily driven by the loss of INO80 itself, rather than compensatory redistribution of P400 or SRCAP. (2) Depletion of P400 leads to a decrease in INO80 chromatin binding. Notably, in regions where both INO80 and P400 binding are significantly altered following P400 degradation, the changes in H2A.Z, H2A.Zac, BRD2, Pol II, and TT-seq signals are more pronounced compared to regions affected solely by P400 depletion. This indicates that INO80 and P400 may exhibit redundant functions at a subset of genes. These findings underscore the complex regulatory network governing the function of these remodelers in the chromatin landscape in mammalian cells.

### Regulatory circuit coordinating BRD2 chromatin occupancy, INO80/SWR remodeler function, and H2A.Z modification states

**Figure 6.**
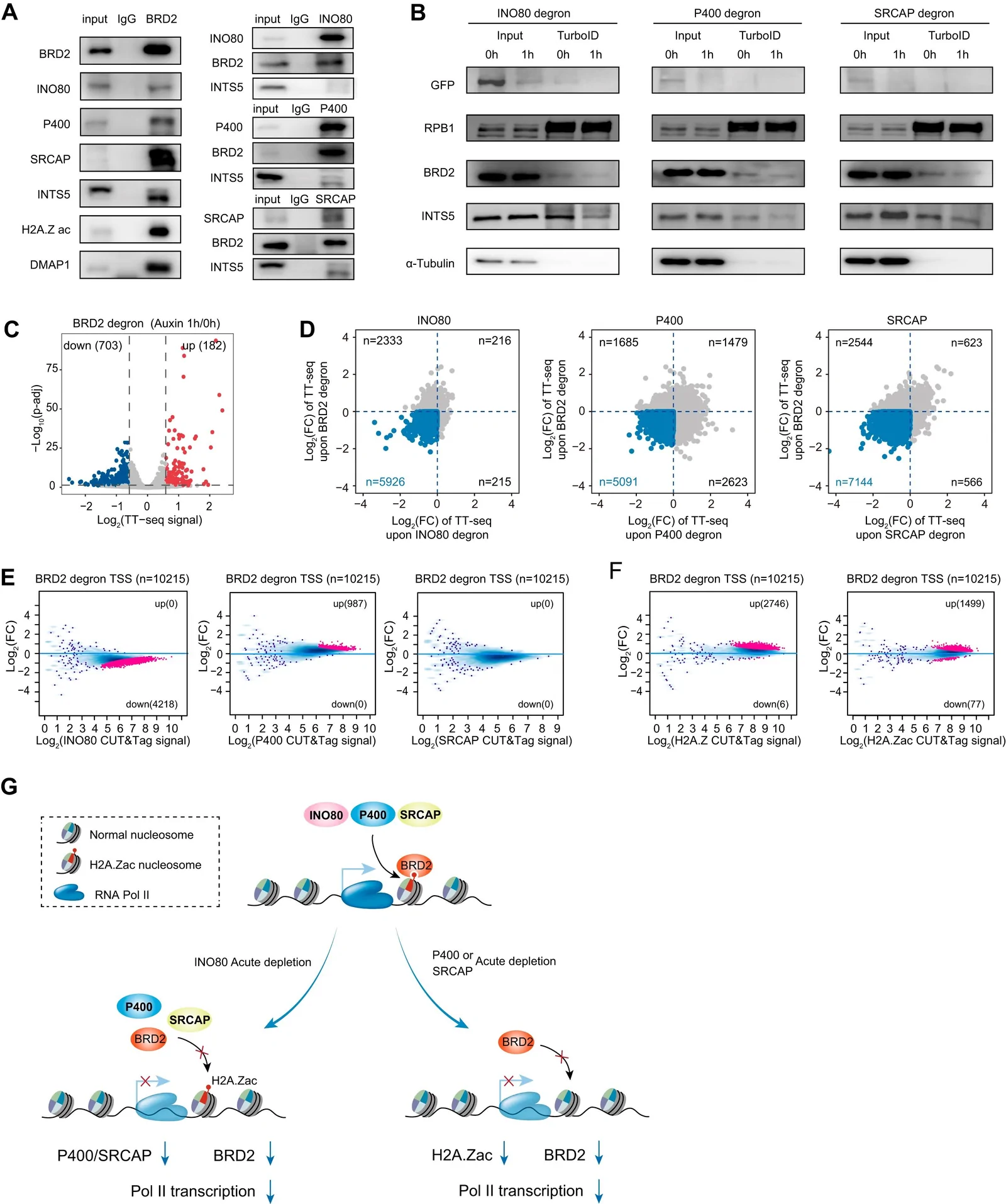
A regulatory circuit coordinates BRD2 occupancy, INO80/SWR chromatin remodeler function, and H2A.Z modification states. (**A**). Co-immunoprecipitation (Co-IP) assay. Left: Lysate of INO80, P400, SRCAP GFP tag degron cells were immunoprecipitated with antibodies against either BRD2 or rabbit IgG. 5% of cell lysate without antibodies was loaded as an input. Western blot analyses of anti-GFP antibody revealed a strong interaction between INO80, P400, SRCAP and BRD2. Western blot using antibodies against DMAP1, INTS5, and H2A.Zac to confirm their interactions with BRD2. Right: Lysate of INO80, P400, SRCAP GFP tag degron cells were immunoprecipitated with antibodies against either GFP (“INO80” lane, “P400” lane, “SRCAP” lane) or rabbit IgG. Western blot analyses of anti-GFP antibody revealed interaction between P400, SRCAP, and INTS5. For BRD2, chromatin was cross-linked, sheared, and immunoprecipitated using an antibody against GFP. Normal IgG and input chromatin served as negative and positive controls, respectively. (**B**). TurboID-Western Blot assay. Western blot showing BRD2 and INTS5-RPB1 associations are reduced after the INO80, P400 and SRCAP degradation. INO80/SWR remodelers (GFP-tagged) do not appear to interact with RPB1 (TurboID-tagged). (**C**). Volcano plots showing the differentially expressed genes identified by TT-seq experiment after 1 h of BRD2 degradation. The differentially expressed genes were identified using DESeq2 software (FDR < 0.05, absolute fold change > 1.5). (**D**). Scatter plot showing the log_2_ fold change log_2_(FC) in TT-seq signals upon BRD2 versus INO80/SWR remodeler degradation. Genes exhibiting significant downregulation (log_2_(FC) < 0 in both conditions) are highlighted in blue, indicating coregulated transcriptional targets. (**E**and **F**). The MA plots show the INO80, P400, SRCAP, H2A.Z, and H2A.Zac CUT &Tag signal changes at TSS regions after degradation of BRD2. Regions with increased or decreased binding were identified by DESeq2 software (FDR < 0.05) and marked red. (**G**). Proposed model of INO80/SWR remodelers regulating gene transcription activation in mammalian cells.

We performed further analyses to gain mechanistic insights into the BRD2-roles in INO80/SWR involved Pol II transcription. We first validated the interactions between BRD2 and INO80, P400, SRCAP, including DMAP1 (a shared subunit of both P400 and SRCAP), INTS5, and H2A.Z acetylation (H2A.Z ac) through BRD2 immunoprecipitation. Furthermore, we confirmed a robust interaction between the INO80/SWR chromatin remodelers and BRD2 using remodeler-specific immunoprecipitation (Fig. 6A). To further validate the Pol II-TurboID mass spectrometry results, we performed Western blot analysis in INO80, P400, and SRCAP GFP-tagged degron with Pol II-TurboID cells (Fig. 6B). The results demonstrated a reduction in BRD2 and INTS5 labeling by Pol II-TurboID upon degradation of INO80, P400, or SRCAP. To functionally dissect the relationship between BRD2 and INO80/SWR remodelers, we performed systematic BRD2 degradation studies (1h). Initial TT-seq comparisons between BRD2 and remodeler degron conditions identified a substantial overlap in downregulated genes, suggesting shared transcriptional regulatory pathways (Fig. 6C and D). Subsequent molecular characterization demonstrated that BRD2 depletion differentially affects the remodelers: while INO80 occupancy decreased and P400 increased, SRCAP occupancy remained stable (Fig. 6E, Supplementary Fig. S7C)(Supplementary Table S4). Importantly, we observed concomitant elevation of both H2A.Z and its acetylated form (H2A.Zac) following BRD2 degradation (Fig. 6F), implying the activation of a compensatory feedback loop that functionally connects BRD2 with H2A.Z chromatin dynamics.

## Discussion

Through generating a large amount of robust dataset (Supplementary Fig. S7D), we identified the direct target genes of the INO80, P400, and SRCAP remodelers in mESCs, providing molecular mechanisms of their specific roles in Pol II transcription. As summarized in Fig. 6G, our findings demonstrate that these remodelers facilitate BRD2 chromatin occupancy, which underpins the mechanism by which INO80/SWR remodelers regulate Pol II transcription. Furthermore, we observed that these remodelers regulate distinct chromatin landscapes; for instance, P400 and SRCAP influence H2A.Z acetylation, while INO80 modulates H3.3 occupancy. Additionally, we investigated the cross-regulations among the INO80/SWR remodelers and found that INO80 facilitates the chromatin binding of P400 and SRCAP, revealing the complex, interconnected functions of these remodelers.

Previous studies have shown that H2A.Z interacts with BRD2, knockdown of H2A.Z decreases BRD2 chromatin binding, and HDAC inhibitor TSA treatment increases the interactions between BRD2 and H2A.Z, while acetylation mutations of H2A.Z did not show such increases. Furthermore, the crystal structures of BRD2 with H2A.Zac have also been reported [103–105]. Together, these results demonstrate that BRD2 directly interacts with H2A.Zac at chromatin in cells. Thus, our results suggest that INO80/SWR remodelers affect H2A.Zac, which would explain the decrease in BRD2 binding. On the other hand, since INO80/SWR remodelers also interact with BRD2, this implies that INO80/SWR may facilitate the chromatin binding of BRD2, specifically for INO80, because INO80 degradation did not affect H2A.Zac levels. BRD2 degradation affects Pol II at a broader set of genes than the remodeler degrons, this observation does not preclude the possibility that BRD2 plays a role in mediating the transcriptional effects of the remodelers at their direct targets. Instead, it highlights the complexity of BRD2’s regulatory network, which may involve both remodeler-dependent and independent mechanisms. Although BRD2 showed similar trend of chromatin binding changes at both remodeler direct target genes and all active genes, the changes at active genes showed less statistical significance compared to those at direct targets. While we acknowledge that transcriptional effects may influence transcription-related factors, the additional evidence we present supports the conclusion that BRD2 plays a unique functional role in this context.

Our study utilized acute protein degradation and subsequent gene expression analyses to reveal the preferential regulation of gene expression by these remodelers, which cannot be easily disentangled using RNAi perturbations or mutagenesis due to potential secondary effects on chromatin structure. We compared our findings with published shRNA-based KD/KO studies [44, 106] (Supplementary Fig. S7E). For SRCAP, both our 1- and 24-h degron depletion and shRNA KO of GAS41 (a subunit of SRCAP) show reduced H2A.Z levels, but the dynamics differ. For INO80, shRNA KO reports decreased Pol II levels, while our 1-h depletion increases Pol II, and 24-h depletion shows no change. There was little correlation between them. These differences highlight that short-term degron depletion captures transient effects distinct from long-term shRNA outcomes. The degradation observed after 1 h reveals immediate effects, albeit limited in scope. Consequently, our definition of direct target genes—characterized by at least a 50% reduction and a two-fold decrease in RNA—may not fully capture a robust phenotype. While longer depletion times could yield more pronounced effects, they may also introduce potential secondary consequences. Therefore, time series analyses of the degron would be critical for gaining deeper insights in the future. We degraded the largest subunits of INO80/SWR remodelers to investigate their functions, and depleting other small subunits may reveal additional roles. While our degron experiments showed immediate effects after remodeler loss, we cannot completely rule out secondary impacts and alternative explanations. Further experiments involving rescue with various mutants would help solidify our conclusions.

While the classical functions of INO80/SWR remodelers are general, their functions likely involve specific factors for different gene expressions. We found that 1 h degradation of INO80 leads to a reduction in both P400 and SRCAP occupancy. INO80 depletion leads to Pol II occupancy changes at thousands of loci while significantly affecting nascent transcription (TT-seq signals) at only a small subset of these (192 genes), suggesting that INO80 may primarily modulate Pol II distribution rather than directly regulating transcriptional output per se at transcription termination sites. Our ChIP-seq data reveal that all three remodelers (INO80, P400, and SRCAP) predominantly localize to transcription start sites. Given this clear enrichment pattern, our study primarily emphasizes their regulatory roles at TSS regions. While we cannot exclude potential effects on Pol II at transcription end sites, these may represent secondary effects of transcriptional changes initiated at transcription start sites or reflect unexplored regulatory mechanisms in other DNA/RNA metabolic pathways that warrant future investigation. For the relationship of chromatin among INO80, SRCAP and P400: Even though the INO80 degron reduces the occupancy of the other two, and P400 degron causes reduced binding of INO80, we cannot directly predict that SRCAP chromatin binding should decrease. There are many protein factors that occupy gene promoters and are directly or indirectly involved in chromatin binding and transcription - not just INO80, P400, and SRCAP. Many other factors may be directly or indirectly involved in remodeler chromatin occupancy.

INO80/SWR remodelers have been known to play a critical role in controlling specific cell states [41, 44, 45, 106]. However, given their general roles in chromatin dynamics, it is unclear how they contribute to the establishment of cell-type-specific gene expression programs. We used an auxin-inducible degron in mESCs cultured in 2i medium, which reflects a naïve pluripotent state. Different protein degradation strategies in other cell types may uncover cell-type-specific functions of INO80/SWR remodelers. While we present a substantial amount of data and report interesting phenomena, further investigation into the underlying detailed mechanisms is warranted. Additionally, the biological functions of this molecular mechanism in relation to gene expression in tissue-specific cell types during development or disease also need to be explored. Specifically, further investigation of INO80/SWR-associated transcription factor motifs or H2A.Z acetylation-mediated regulation may uncover additional biological functions of INO80/SWR in transcriptional regulation within specific cell lineages. Furthermore, the regulatory landscape beyond histone acetylation—such as other histone modifications or unexplored regulators—may also play a role in the mechanisms by which INO80/SWR regulates Pol II transcription in mammalian cells.

## Supplementary Material

gkaf892_Supplemental_Files

## Data Availability

All the data that support the findings of this study are available from the corresponding authors upon reasonable request. The raw sequencing data can be found: GEO: GSE237534. The raw mass spectrometry data can be found: PRIDE database (Project accession: PXD045590). All raw Western blotting images and other relevant image data to the Mendeley Data repository (DOI: 10.17632/nzkv23hz3p).
