# Correction to ‘INO80/SWR remodelers regulate Pol II transcription through BRD2 and chromatin landscape’

**DOI:** 10.1093/nar/gkag913

**Published:** 2026-09-15

This is a correction to: Meitong Liu, Qiqin Xu, Hui Wang, Xiong Ji, INO80/SWR remodelers regulate Pol II transcription through BRD2 and chromatin landscape, *Nucleic Acids Research*, Volume 53, Issue 17, 23 September 2025, gkaf892, https://doi.org/10.1093/nar/gkaf892

Two clerical/copying errors were identified in Tables S1 and S2.

Table S1: a formula error occurred during data export. Three mapping ratios were inadvertently pasted as identical static values rather than their actual calculated formulas. This resulted in the duplication of mapping rates for three samples: ATAC-seq_INO80_degron_1h_rep1, ATAC-seq_INO80_degron_1h_rep2, and ATAC-seq_INO80_degron_0h_rep1.

Table S2: due to a copy-paste oversight, the mass spectrometry data for Chromatin-MS upon SRCAP degradation was accidentally duplicated from the Chromatin-MS upon INO80 degradation dataset.

Tables S1 and Table S2 have been corrected. In the revised Table S1, the mapping ratios now display the correct calculated values. In the revised Table S2, the erroneous SRCAP degradation data has been replaced with the accurate experimental results.

All raw data were correctly generated and deposited prior to publication; these changes strictly address formatting errors in the tables and do not affect the scientific conclusions of the study.

