# Supplementary material for: INO80/SWR remodelers regulate Pol II transcription through BRD2 and chromatin landscape
Source: Nucleic Acids Res. 2025 Sep 17;53(17):gkaf892. doi: 10.1093/nar/gkaf892 (PMC12448918; doi:10.1093/nar/gkaf892)
Supplement: gkaf892_Supplemental_Files [file gkaf892_supplemental_files.zip › Supplementary Figures_20250716.pdf]

Figure S1

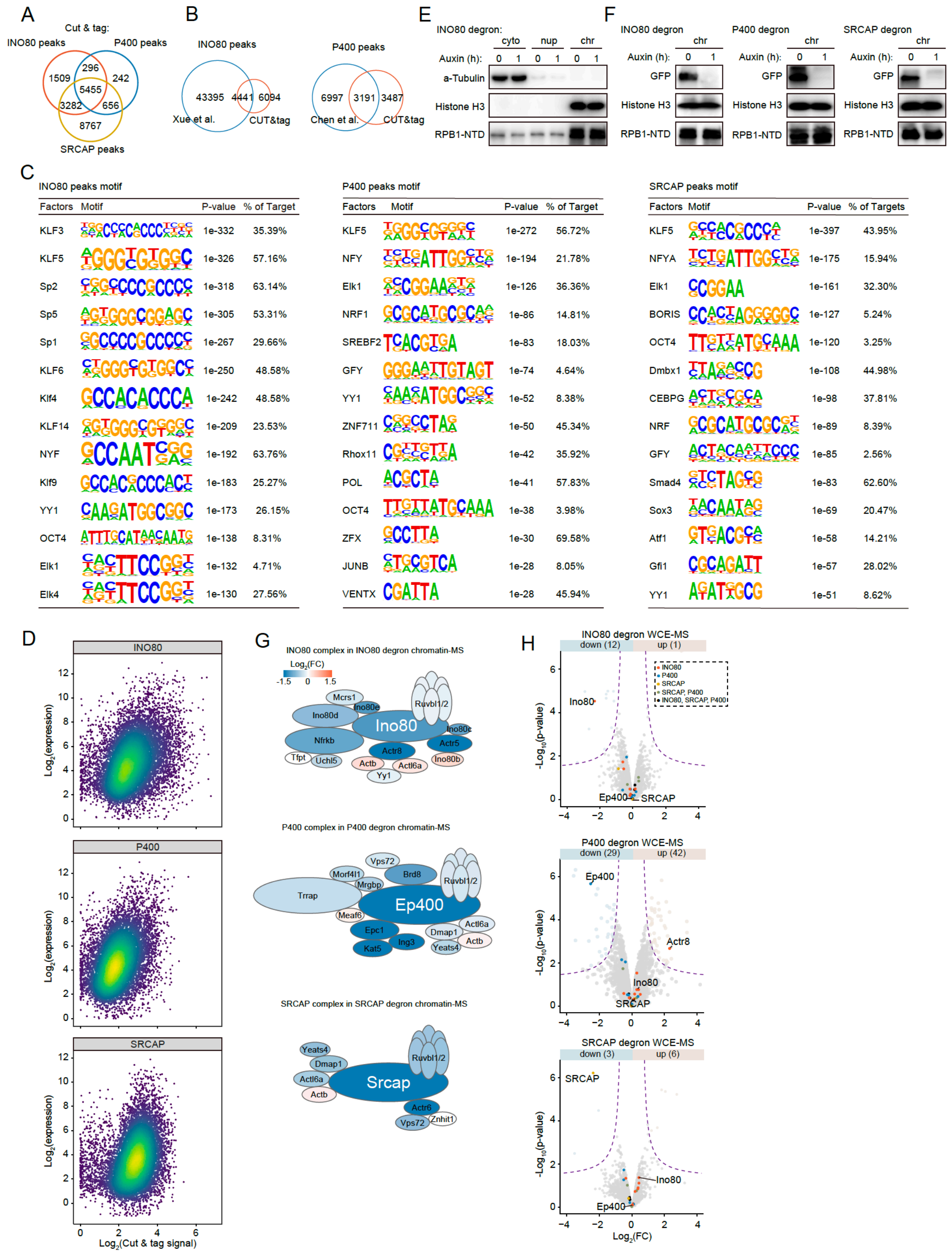

Figure S1. Validation of the degron systems for INO80/SWR remodelers.

A. Overlap of the CUT&Tag peaks of INO80, P400 and SRCAP with GFP antibodies and degron-GFP mES cells.

B. Venn diagram showing the overlap between INO80 or P400 peaks identified in our study and peaks identified by published data. The peaks of published data were obtained from Cistrome database.

C. Representative motifs of the INO80/SWR remodelers.

D. The binding of INO80, P400, and SRCAP shows a positive correlation with

gene expression. The correlation coefficients for INO80, P400, and SRCAP are 0.34, 0.38, and 0.3, respectively.

E. Western blot assays the purity of the isolated chromatin.
F. Western blot to examine the degradation of the target proteins.
G. Chromatin-MS results revealing the obvious degradation of the target protein (mAID-fused), accompanied by either degradation or disassembly of other subunits from chromatin. The color indicates the fold change in abundance, while the size represents the relative molecular weight of the subunit.
H. Volcano plot showing the proteins with altered abundance in Whole cell extract (WCE)-MS upon INO80 and SRCAP degradation.

Figure S2

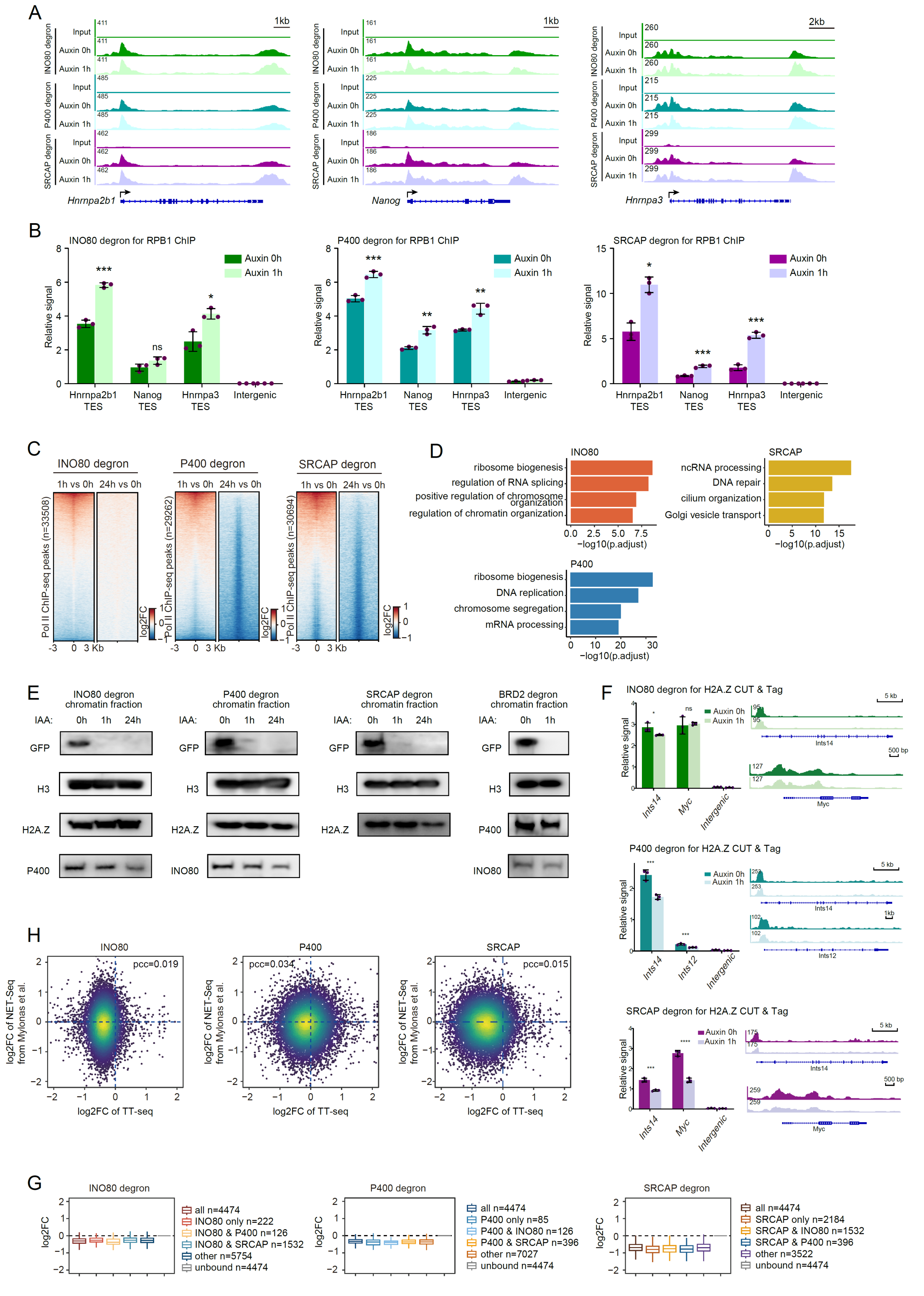

Figure S2. Depletion of INO80/SWR remodelers affects Pol II binding and H2A.Z occupancy.

A. Representative gene tracks of the Pol II binding enhanced at TES regions after the depletion of the INO80/SWR remodelers.

B. ChIP-qPCR was conducted to investigate the changes in Pol II occupancy

at the gene transcription end site (TES). The experiment included at least two replicates, and the error bars represent the standard deviation. The significance of the results was determined using the student's t-test, and the p-values were calculated. The notation used for statistical significance is as follows: Ns (not significant), * ($p<0.05$), ** ($p<0.01$), *** ($p<0.001$).

C. Heatmap showing log2 fold change of Pol II signal at genes following 1 hour and 24 hours degradation of INO80, P400, and SRCAP.
D. Gene ontology analysis of INO80, P400 and SRCAP's direct target genes.
E. Western blot analysis of chromatin fractions isolated from cells following inducible degradation of the remodeler and BRD2 (via degron system). The blot demonstrates changes in chromatin-associated H2A.Z levels upon remodeler depletion and reciprocal regulatory effects between INO80 and P400 on their respective chromatin binding. Additionally, BRD2 appears to modulate the chromatin association of both P400 and INO80. Histone H3 serves as a loading control for chromatin-bound proteins.
F. Representative gene tracks of the H2A.Z at TSS regions after the depletion of INO80/SWR remodelers and CUT&Tag qPCR validation of the changes of them on chromatin. The experiment included at least two replicates, and the error bars represent the standard deviation. The significance of the results was determined using the student's t-test, and the p-values were calculated. * $p< 0.05$, ** $p< 0.01$, *** $p< 0.001$, **** $p< 0.0001$.
G. Boxplots showing the log2 fold change of H2A.Z CUT&Tag signal upon the degradation of INO80, P400 or SRCAP at genes bound by one, two or three remodelers. The unbound genes were selected as firstly the genes with no remodeler peaks and then the genes with least remodeler binding signal. The unbound genes were selected to the same number of the genes bound by all the three remodelers.
H. Dot plots showing the correlation of the changes of TT-seq upon the degradation of INO80, P400 and SRCAP and the changes of NET-seq after the degradation of H2A.Z. The NET-seq data were downloaded from previous publication.

Figure S3

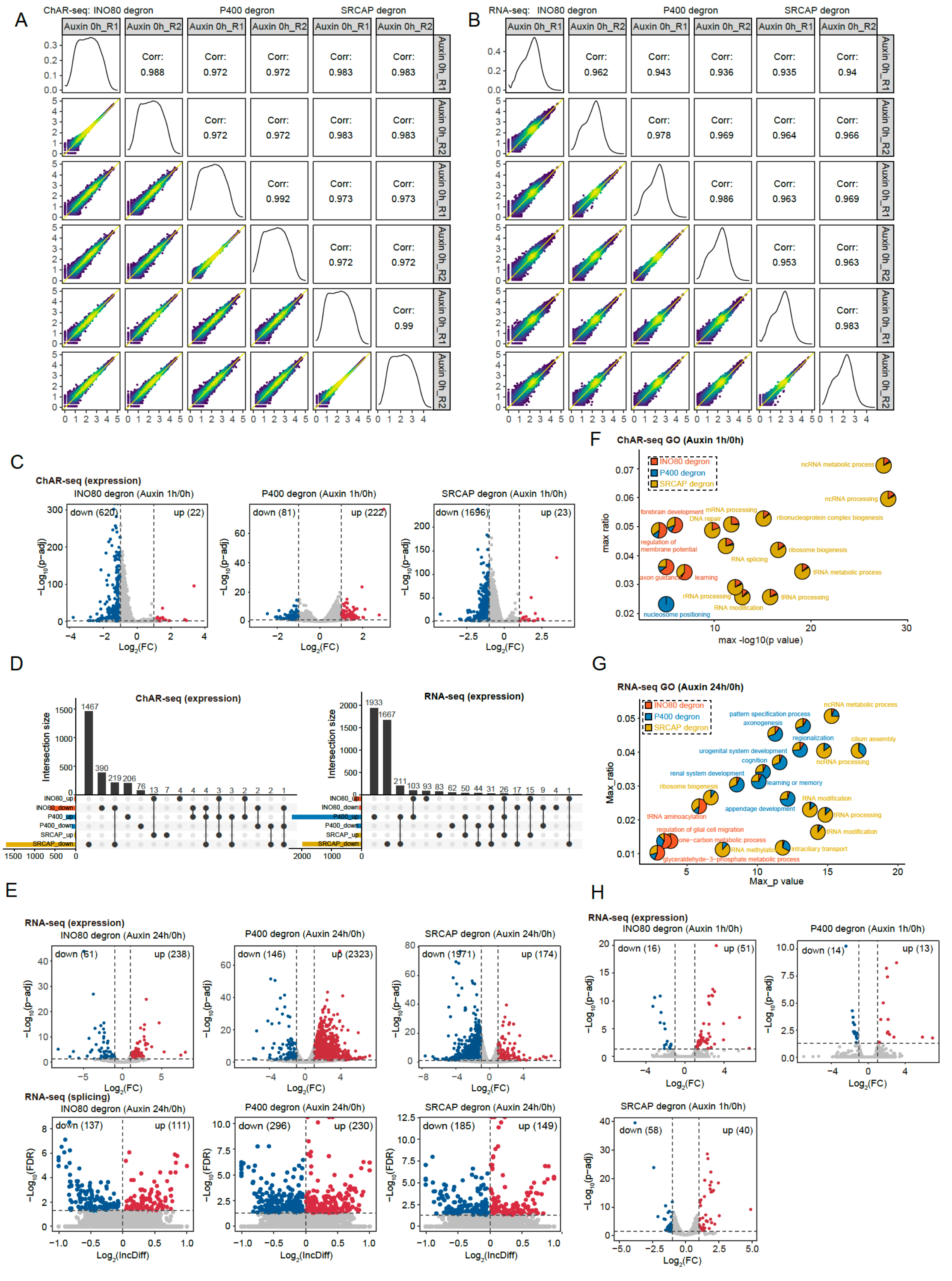

Figure S3. Depletion of INO80/SWR remodelers leads to distinct dysregulations in gene expression.

A-B Correlation plots of ChAR-seq (A) and RNA-seq (B) expressions of all the genes in INO80, P400 and SRCAP degron cells under untreated conditions.

C&E Volcano plots showing the differentially expressed genes identified by ChAR-seq (C) or RNA-seq (expression and splicing) (E) experiment upon INO80, P400 and SRCAP degradation. The differentially expressed genes

were identified using DESeq2 software (FDR < 0.05, log2FC > 1).

D. Upset plots showing the overlap of differentially expressed genes identified by ChAR-seq (D) or RNA-seq (F) experiment.

F-G. GO enrichment analysis for the differentially expressed genes (DEG) identified by ChAR-seq (F) and RNA-seq (G). Each pie shows a GO term and indicates the proportion of the enriched DEGs under the three different perturbations. The proportion of DEGs mapped on a term were adjusted for duplicated genes and gene sizes in a same way as ClueGO did. The location of each pie was displayed using the p-value and gene ratio of DEGs in that term with the largest proportion.

H. Volcano plots showing the differentially expressed genes identified by RNA-seq experiment after 1 hour of INO80, P400 and SRCAP degradation. The differentially expressed genes were identified using DESeq2 software (FDR < 0.05, log2FC > 1).

Figure S4

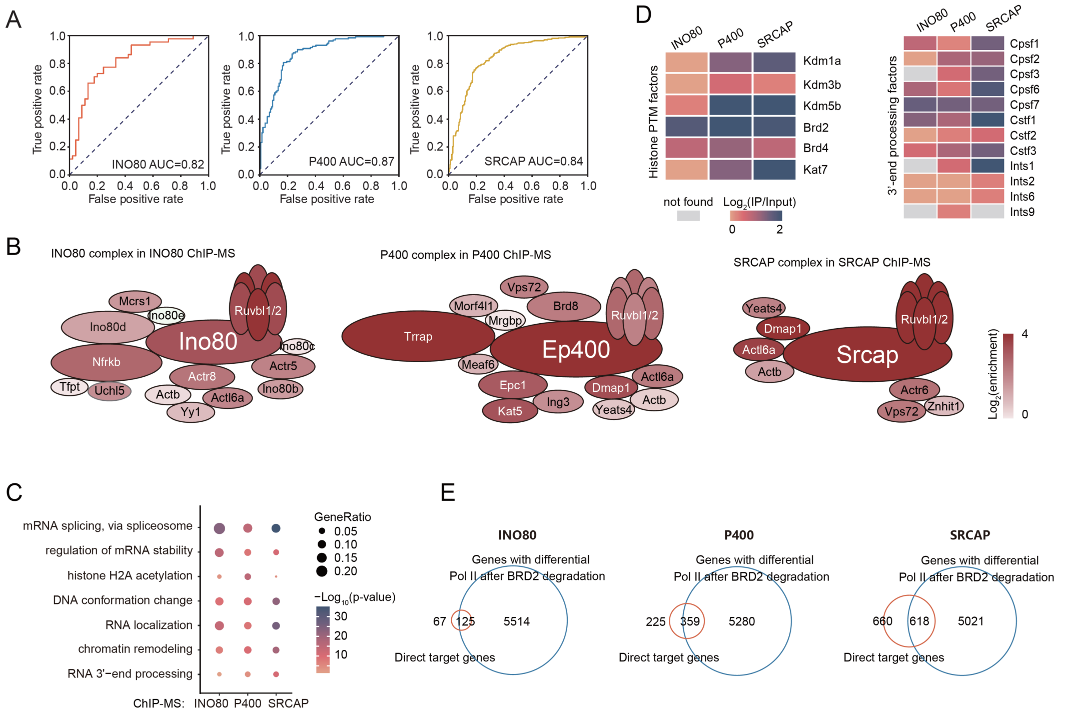

Figure S4. INO80/SWR remodelers are associated with histone PTM factors and RNA 3'end processing factors.

A. ROC curves of the GLM models.

B. ChIP-MS results showing the enrichment of subunits in INO80, P400 or SRCAP complex. The color indicates log2 IP/Input. The circle size represents the relative molecular weight of the subunit.

C. GO enrichment analysis for the enriched proteins identified in INO80, P400 or SRCAP ChIP-MS. Proteins with log2 IP/Input > 1 were used for analysis.

D. Heatmap showing the log2 enrichment of several histone PTM factors (left) and 3'-end processing factors (right) in INO80, P400 and SRCAP ChIP-MS.

E. Venn diagram showing the overlap between direct target genes of INO80, P400, SRCAP and the genes with differential Pol II binding after 3 hours of BRD2 degradation, a dataset published previously (98).

Figure S5

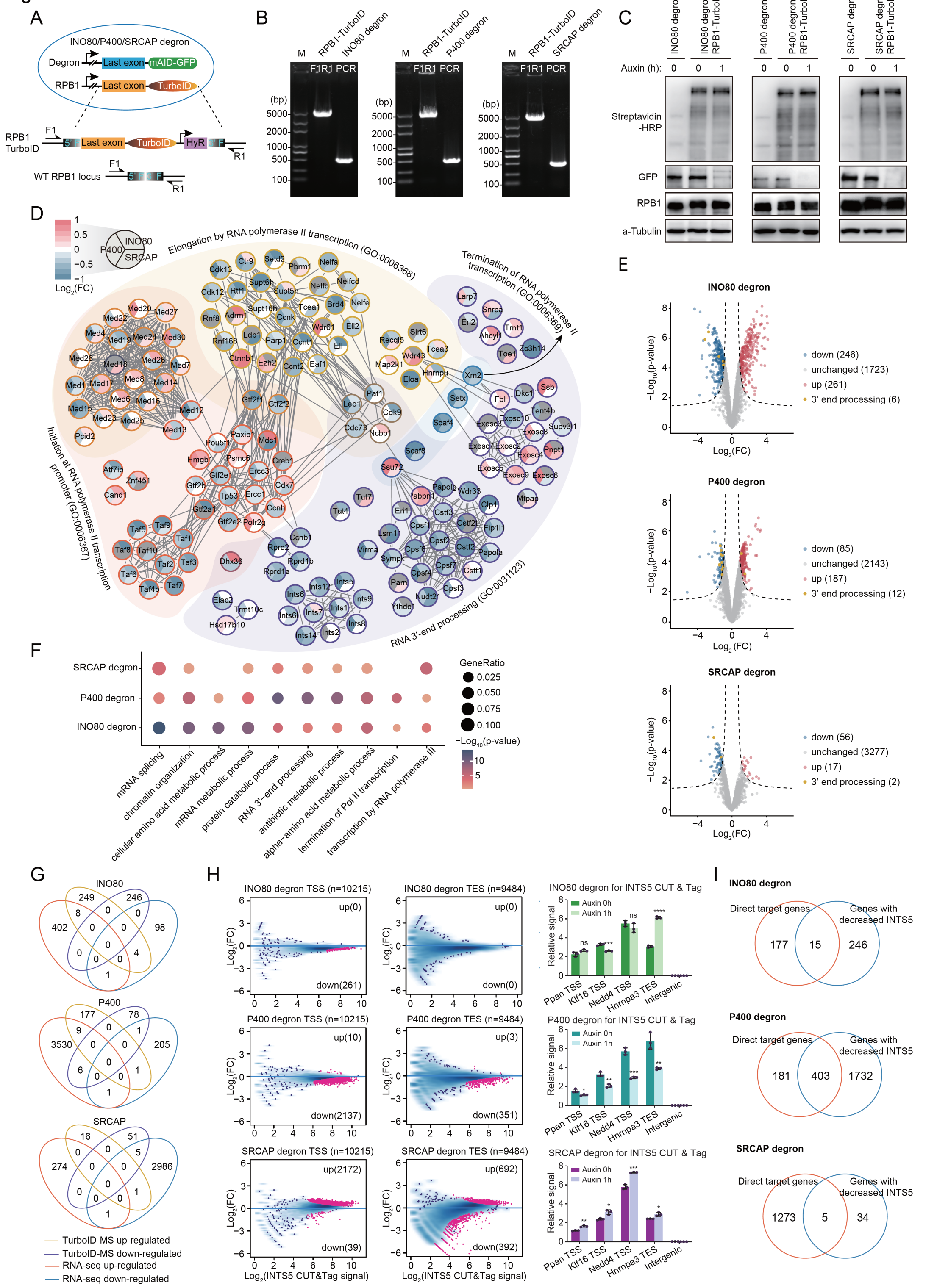

Figure S5. Many RNA metabolic factors changed interactions with Pol II after the degradation of INO80/SWR remodelers.

A. Diagram model of the RPB1-TurboID in the three remodelers degrons.
B. Genotyping assay the homologous of the RPB1-TurboID knock-in sites.
C. Western blot to examine the biotin labeling efficiency, no TurboID cells were used as negative control.

D. Changes in interactions of transcription initiation, elongation, termination, and 3' end processing factors during the degradation of INO80/SWR remodelers.
E. Volcano plot depicting the differentiation interactome of Pol II before and after degradation of remodelers, as identified through TurboID labeling.
F. Gene ontology (GO) analysis of the proteins differentially affected in the TurboID-identified interactome.
G. Venn diagrams showing the overlap among up or down regulated proteins identified by TurboID-MS and RNA-seq upon the degradation of INO80, P400 and SRCAP.
H. Left: The MA plots show the INTS5 CUT&Tag signal changes at TSS or TES regions after degradation of the INO80/SWR remodelers. Regions with increased or decreased INTS5 binding were identified by DESeq2 software (FDR < 0.05) and marked red. Right: CUT&Tag-qPCR validation the changes of INTS5 chromatin occupancy. The experiment included at least two replicates, and the error bars represent the standard deviation. The significance of the results was determined using the student's t-test, and the p-values were calculated. * $p< 0.05$, ** $p< 0.01$, *** $p< 0.001$, **** $p< 0.0001$.
I. Venn Diagram showing the overlap of genes with decreased Pol II signal at TSS regions and unaltered Pol II signal at TES regions, genes with unaltered Pol II signal at TSS regions and increased Pol II signal at TES regions, genes with changed INTS5 signal at TSS and TES regions upon INO80, P400 or SRCAP degradation.

Figure S6

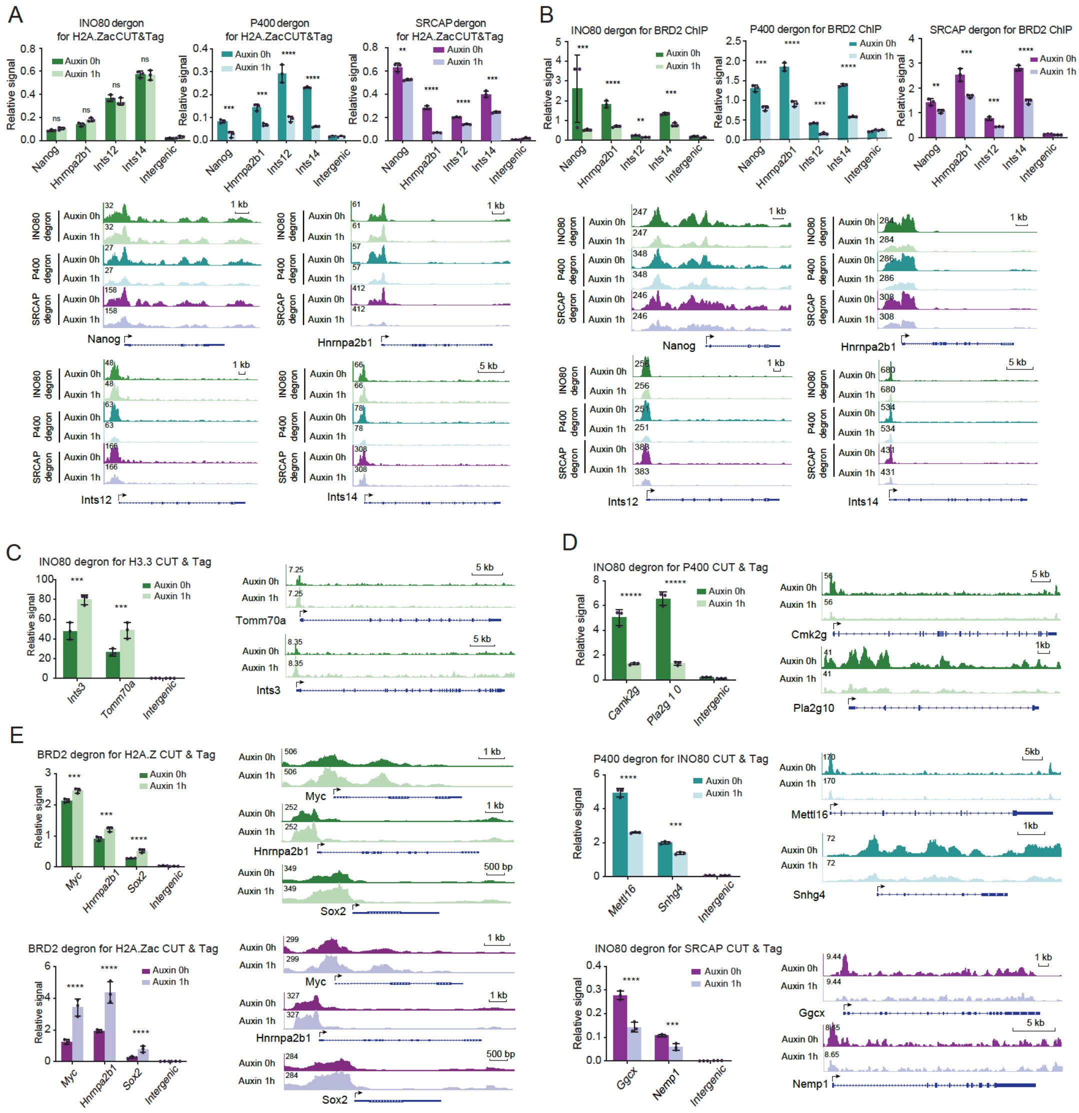

Figure S6. The INO80/SWR affected H2A.Z acetylation, H3.3, and the binding of other INO80/SWR remodelers.

A-C. Representative gene tracks of the H2A.Zac, BRD2 and H3.3 at TSS regions after the depletion of the INO80/SWR remodelers and CUT&Tag-qPCR and ChIP-qPCR validation of the changes of them on chromatin. The experiment included at least two replicates, and the error bars represent the standard deviation. The significance of the results was determined using the student's t-test, and the p-values were calculated. * p< 0.05, ** p< 0.01, *** p< 0.001, **** p< 0.0001.

D. Representative gene tracks and CUT &Tag- qPCR validation of the remodelers cross-regulation.

E. Representative gene tracks and CUT&Tag- qPCR validation of H2A.Z and H2A.Zac at TSS regions after the depletion of BRD2 and CUT &Tag- qPCR validation of the changes of them on chromatin.

Figure S7

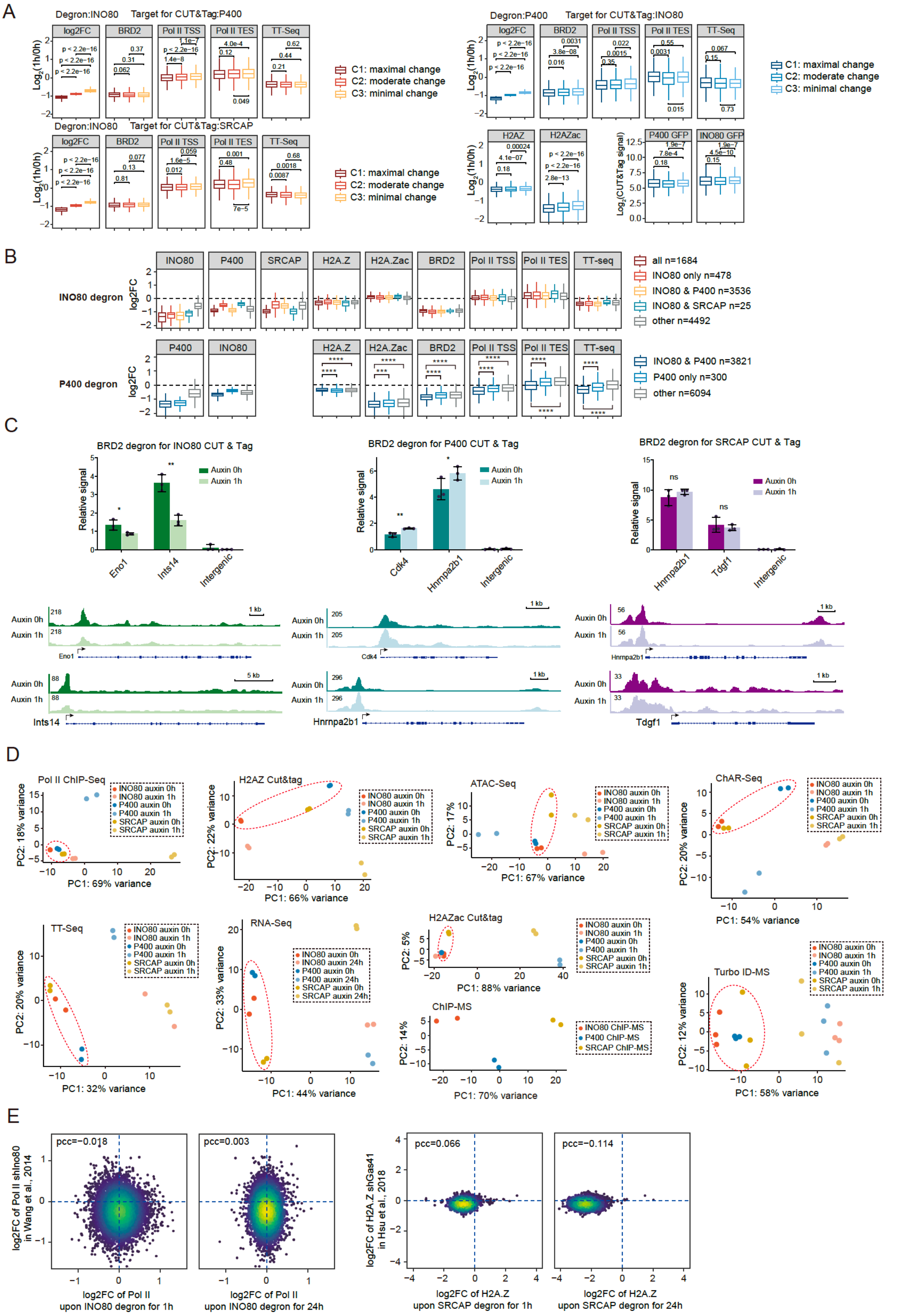

Figure S7. Validation of cross-regulatory interactions, global PCA analysis, and representative gene tracks.

A. Top three lines: Boxplots showing the log2 fold change of P400, SRCAP or SRCAP, BRD2, Pol II TSS and TES ChIP-Seq signals and TT-Seq signals upon the degradation of INO80 or P400 at three gene clusters. The genes

are classified into maximal, moderate and minimal changes according to the log2 fold change of P400, SRCAP or SRCAP CUT&Tag signals upon the degradation of INO80 or P400. Last line: Left two boxplots show the log2 fold change of H2AZ and H2AZac signals upon the degradation of P400 at three gene clusters. Right two boxplots show the P400 and INO80 CUT&Tag signals at the three gene clusters. Statistical analysis was determined using the Wilcoxon test.

B. Boxplots showing the log2 fold change of INO80, P400, SRCAP, H2A.Z, H2A.Zac CUT &tag, BRD2, Pol II ChIP-seq or TT-seq upon the degradation of INO80 (upper panel) or P400 (bottom panel). Statistics were performed using Wilcoxon test (*** $p < 0.001$, **** $p < 0.0001$).

C. Representative gene tracks and CUT&Tag- qPCR validation of the INO80/SWR remodelers at TSS regions after the depletion of BRD2.

D. PCA plots of Pol II ChIP-seq, TT-seq, RNA-seq, H2A.Z CUT &Tag, H2A.Zac CUT &Tag, ATAC-seq, ChAR-seq, TurboID-MS and ChIP-MS. The same color represents experimental repeats. The red dashed line indicates the untreated control group.

E. Scatter plot shows the correlation analysis of log2FC of Pol II or H2A.Z after the degradation of INO80 or SRCAP for 1h (left) and 24h (right), and log2FC of Pol II or H2A.Z after shIno80 or shGas41.
